# Three joint temperament-character configurations account for learning, personality and well-being: normative demographic findings in a representative national population

**DOI:** 10.3389/fpsyg.2023.1193441

**Published:** 2023-07-03

**Authors:** Paulo A. S. Moreira, Richard A. Inman, C. Robert Cloninger

**Affiliations:** ^1^Centro de Investigação em Psicologia para o Desenvolvimento (CIPD), Lisbon, Portugal; ^2^Instituto de Psicologia e Ciências da Educação, Universidade Lusíada, Porto, Portugal

**Keywords:** personality, temperament profiles, character profiles, temperament-character configurations, representative national population, epidemiology, personality and demographic interactions

## Abstract

**Introduction:**

A common practice in research and clinical practice is to use data considered representative of a target population to compare and understand the personality characteristics of specific groups or specific individuals. To this end, numerous studies have presented normative data for the temperament and character traits outlined in Cloninger’s psychobiological model of personality. However, recent genomic evidence demonstrates that human personality is organized as a complex hierarchy that ascends beyond the individual traits to multi-trait profiles that regulate emotional reactivity (temperament profiles) or goals and values (character profiles), and then to three phenotypic networks, which integrate temperament profiles and character profiles, that regulate learning. Given this recent understanding, our aim was to provide a novel and more comprehensive description of personality features at a societal level (using a stratified sample representative of the Portuguese population) by considering personality at its higher levels of complexity.

**Methods:**

Toward this goal, a stratified sample of 2,443 Portuguese adults responded to the Revised Temperament and Character Inventory (TCI-R).

**Results:**

We summarize the prevalence of (a) temperament profiles, (b) character profiles, and (c) integrated temperament-character networks within the whole sample, as well as for men vs. women and different age groups separately. Independent of age and education, women were more likely to be capable of resourceful productivity and helpful cooperation combined with being more intuitive, meditative and creative than men. Independent of age and gender, individuals with a degree were also more likely to present these biopsychosocial features. We also found that the organized character profile was most typical of adults in their 40s. Finally, the distribution of personality profiles across age differed as a function of gender: for men the oldest individuals had the most coherent personalities while high personality integration was most prevalent for women in their 30s.

**Discussion:**

These results have strong implications for research and intervention. In particular, these results are relevant for understanding the epidemiology of interactions between personality, mental health and well-being, including their expressions in a national population as a function of demographic characteristics.

## 1. Introduction

The revised Temperament and Character Inventory (the TCI-R; Cloninger, 1999) provides a reliable quantitative self-report assessment of the four temperament traits and three character traits described by Cloninger’s psychobiological model of personality (Cloninger, 2004). Reflecting its popularity and strong influence in generating research, the TCI-R has been translated into over 20 languages (e.g., Pélissolo et al., 2005; Goncalves and Cloninger, 2010; Moreira et al., 2012, 2017), and has found to have at least as good (and often stronger) predictive validity than other modern personality inventories (Grucza and Goldberg, 2007).

Recent studies have used various versions of the TCI-R to describe and understand how the personality features of specific groups of people vary relative to the general population (Svrakic et al., 1993; Sievert et al., 2016; Moreira et al., 2019, 2022). Such research is useful because the neurobiologically-grounded traits of Cloninger’s psychobiological model allow for causal explanations of experiences and actions at the individual level (Cervone, 2005). However, for any meaningful interpretation of how the personality features of a subgroup or individual differ from normality it is necessary to have reliable normative data from a reference population. To this end, several studies have presented normative values for individual TCI-R traits and their facets (Moreira et al., 2023a,b).

However, robust genomic evidence has demonstrated that human personality is organized as a complex hierarchy that ascends beyond the individual traits to multi-trait profiles that regulate emotional reactivity (temperament profiles) or goals and values (character profiles), and then to three integrating temperament-character networks that express major systems of learning and memory (Cloninger and Zwir, 2018, 2022; Zwir et al., 2020a,b, 2021, 2022). In light of this evidence, the purpose of the present article was to describe the prevalence of these more complex organizations of personality in a large and representative sample of Portuguese adults.

### 1.1. Temperament profiles

Firstly, the psychobiological model of personality posits a domain of relatively stable aspects of personality – temperament - that captures heritable individual differences in dispositional tendencies to automatically and unconsciously react emotionally, act, and form attachments (Cloninger and Zwir, 2018, 2022; Cloninger et al., 2019; Zwir et al., 2020b). Individual differences in temperament are quantified in terms of four dimensions that are neurophysiologically, neuroanatomically and biochemically distinct from one another: novelty seeking (impulsive and excitable vs. deliberate and reserved), harm avoidance (pessimistic and fearful vs. optimistic and risk-taking), reward dependence (sentimental and approval-seeking vs. objective and independent), and persistence (determined and ambitious vs. easily discouraged and underachieving) (Gusnard et al., 2003; Krebs et al., 2009; Zwir et al., 2020b; Cloninger and Zwir, 2022). All people have some latent level of each dimension, with both advantages and disadvantageous to the extremes of each depending on context, resulting in a high level of variability in temperament in the population.

**Figure 1:**
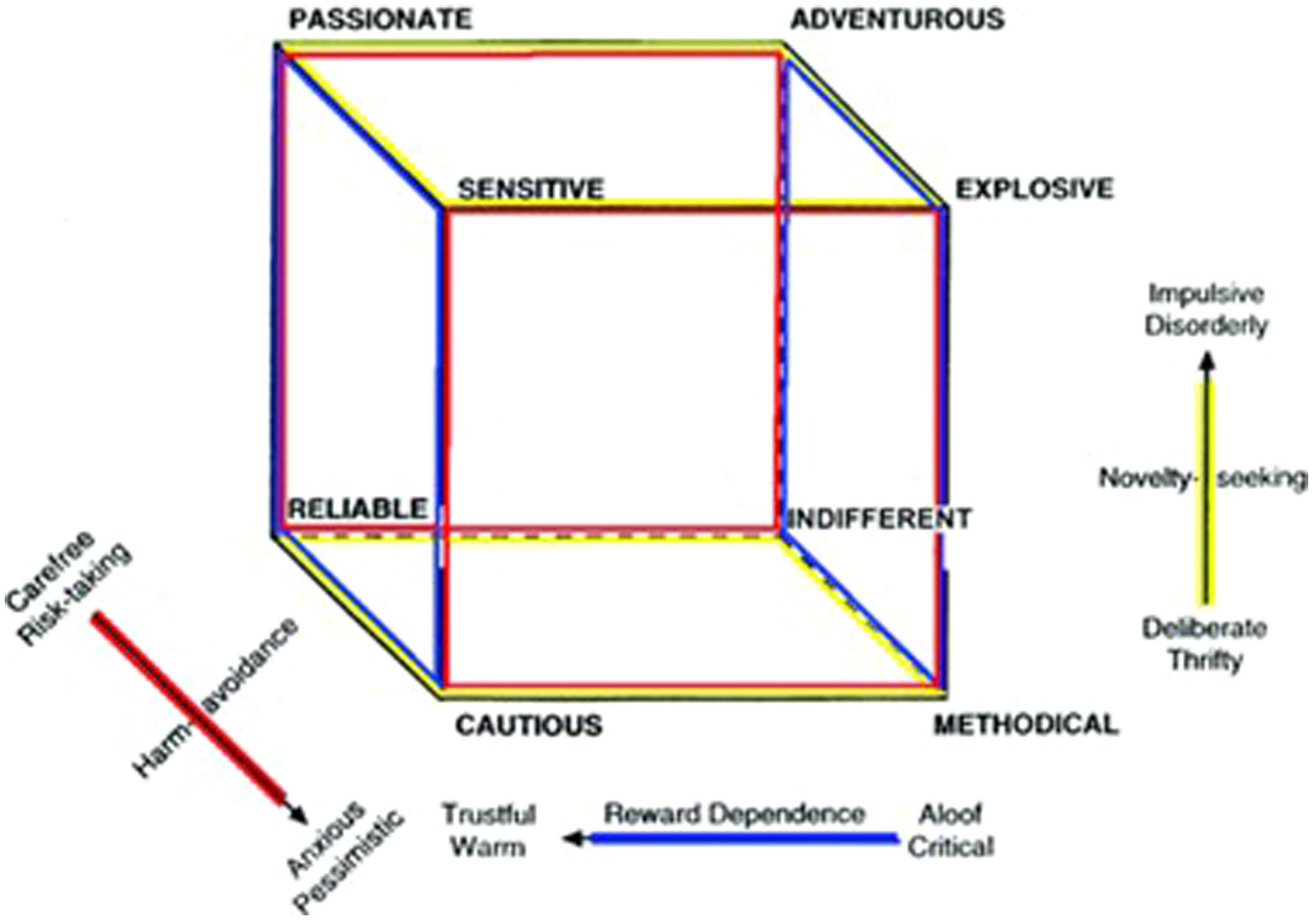
The temperament cube (Reproduced with permission of the copyright holder, Anthropedia Foundation).

Early clinical research showed that different clusters of personality disorders could be distinguished by these temperament dimensions: impulsive personality disorders (DSM Cluster B) linked to high novelty seeking; anxious-fearful personality disorders linked to high harm avoidance (DSM Cluster C); and aloof personality disorders linked to low reward dependence (DSM Cluster A) (Cloninger, 1987; Svrakic et al., 1993; Goldman et al., 1994; Mulder and Joyce, 1997). As such, configurations of novelty seeking, harm avoidance and reward dependence were presented as (and have been long used as) a reliable way to subtype personality disorders (Cloninger, 2000) (see Figure 1). However, more recent research has demonstrated that heritability in temperament depends on genes that modulate molecular processes for associative conditioning and that developed early in the human evolutionary lineage (Cloninger and Zwir, 2022), and that these genes code for different configurations of all four temperament dimensions (Cloninger and Zwir, 2018; Cloninger et al., 2019). In these profiles, individual differences in persistence (high vs. low) quantify the degree to which a person’s habits and emotional tendencies are persistently regulated by the character traits to be coherent with goals and values (Moreira et al., 2022c).

With the aim of evaluating the interactions among specific combinations of temperament traits related to human functioning and wellbeing, several recent studies have adopted person-centered approaches, clustering and comparing groups of individuals with similar temperament profiles. In many of these works, temperament profiles were formed by dividing the sample into participants above and below the median for novelty seeking (N vs. n), harm avoidance (H vs. h), reward dependence (R vs. r) and persistence (P vs. p) and then forming groups according to all 16 theoretically possible combinations of high and low temperament scores. Research comparing groups of people with different temperament profiles has found differences in average presentation of psychopathology, with configurations featuring high novelty seeking and low reward dependence and persistence (as well as slightly higher harm avoidance) linked to increased behavioral and emotional problems (Rettew et al., 2008; Moreira et al., 2021a,b,c,d). Consistent with this work, an explosive temperament profile (NHr) with low persistence was found to occur more frequently in a prisoner population than the general population (Moreira et al., 2022d). In contrast, configurations featuring high reward dependence and persistence with low novelty seeking and/or low harm avoidance have been linked to a lower average probability of psychopathology (Rettew et al., 2008; Moreira et al., 2021a,b,c,d). Even within psychiatric samples, groups of patients with this type of temperament configuration were much more likely to be classed as having no subjective distress (Choi and Lee, 2022). Other comparisons of groups of people with different temperament profiles has also shown that they differ in their average positive traits. For example, those with a methodical (nHr) temperament profile with high persistence tended to score highest for self-control, while others with an adventurous temperament profile with high persistence (Nhr) tended to score highest in inquisitiveness (Moreira et al., 2022c). Further, individuals with a reliable temperament profile (nhR) and high persistence tended to report the highest subjective wellbeing, while those with an explosive temperament profile with low persistence tended to report the lowest subjective wellbeing. Validating the median-split approach to temperament profile formation, various studies employing mixture models or machine-learning algorithms have extracted similar latent temperament profiles, and shown that these differ in a theoretically consistent way in terms of well-being and human functioning (Moreira and Inman, 2021; Zwir et al., 2021; Moreira et al., 2021a,b,c,d; Moreira and Inman, et al., 2022a,b).

### 1.2. Character profiles

Although groups of people with different temperament profiles differ in their average probabilities of having a personality disorder (vs. a mature and healthy personality) these average between-person effects alone were found to be uninformative for distinguishing the maturity or health of any specific individual (Cloninger et al., 1997). In order to do so, the psychobiological model was expanded to include a regulatory and cognitive domain of personality, referred to as character, which recent research has confirmed is genetically, psychologically, and developmentally separate from temperament (Zwir et al., 2020a), but similarly as heritable (Gillespie et al., 2003; Cloninger and Zwir, 2022). Human character reflects organizations of socio-cognitive processes for intentionality and self-awareness that serve functions that are intrapersonal (e.g., planning and foresight), legislative (e.g., empathy and norms for cooperation), and judicial (e.g., insight and intuitive evaluation of what is meaningful and good). Individual differences in these functions are quantified in terms of three dimensions for which all people have some latent value: self-directedness (responsible and purposeful vs. blaming and aimless), cooperativeness (tolerant and empathetic vs. prejudiced and self-centered) and self-transcendence (altruistic and spiritual vs. individualistic and skeptical). Critically, individual differences in these three character dimensions are what distinguish individuals with personality disorders from those without, with low self-directedness being a particularly strong indicator (Svrakic et al., 1993).

**Figure 2:**
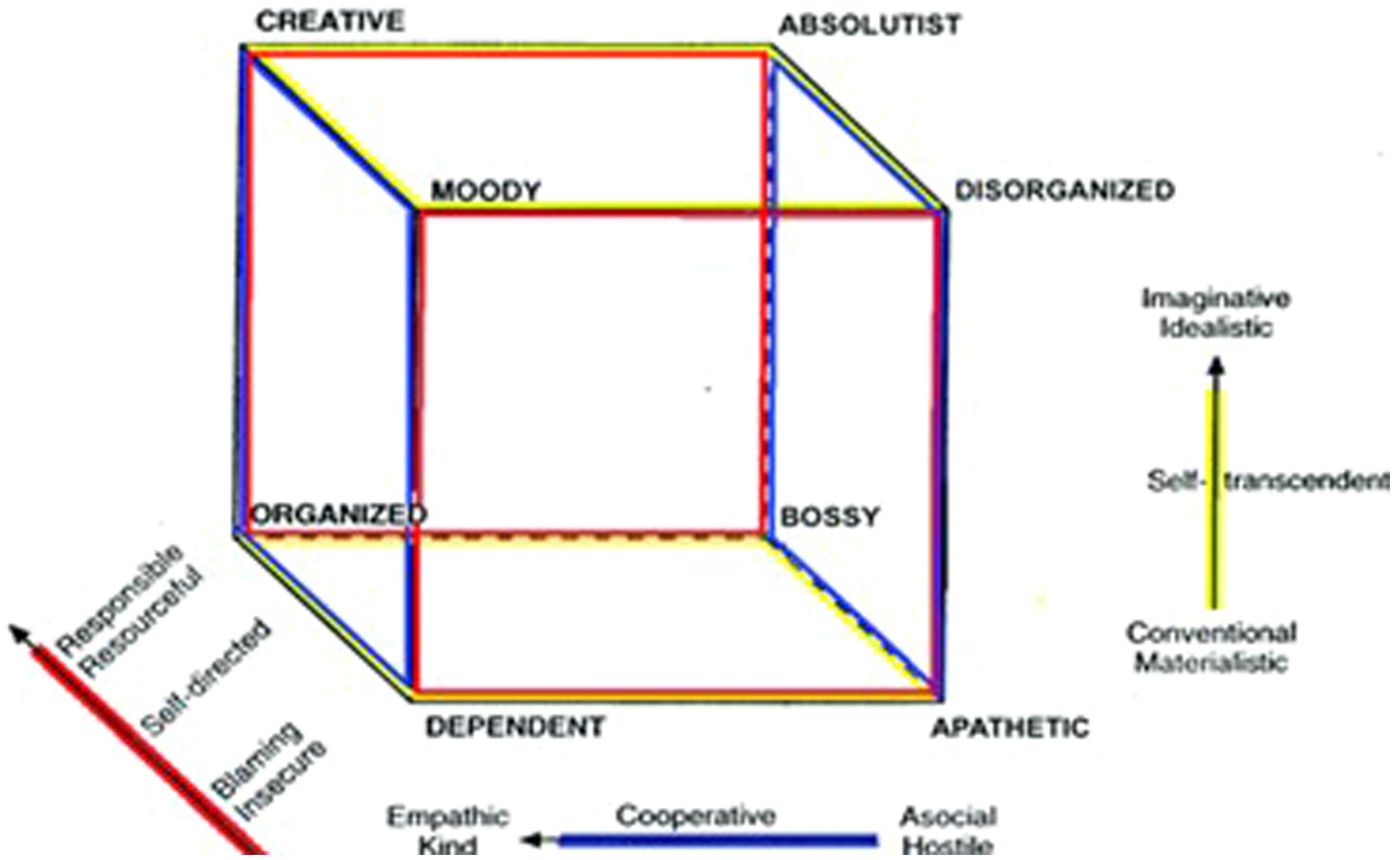
The character cube (Reproduced with permission of the copyright holder, Anthropedia Foundation).

Like for temperament, various studies have explored how interactions among specific combinations of character dimensions relate to human functioning and wellbeing. Similarly, for many works (e.g., Cloninger and Zohar, 2011; Moreira et al., 2015) character profiles were formed by dividing the sample into participants above and below the median for self-directedness (S vs. s), cooperativeness (C vs. c) and self-transcendence (T vs. t). This results in eight theoretical configurations of self-directedness, cooperativeness and self-transcendence, which can be depicted as the corners of a character cube (Cloninger, 2004; see Figure 2). Supporting their naturalistic occurrence, all eight of these profiles have emerged from latent profile analyses (Moreira et al., 2022a).

Various person-centered studies have demonstrated that physical, mental and social well-being is strongly dependent on one’s configuration of character traits (Moreira et al., 2008). From the eight possible profiles, the least healthy people typically have a profile with low self-directedness and cooperativeness. Consistent with this, research has shown that such character profiles are prevalent in prison inmates (Moreira et al., 2022a,b,c,d) and linked to elevated levels of emotional/behavioral problems (Lee et al., 2018; Moreira et al., 2021a) and psychological reactance (Moreira et al., 2022b).

In contrast, profiles with high self-directedness and high cooperativeness describe people who are relatively healthy, although within this group of people those who also have high self-transcendence (the creative character) are typically happier, healthier, and more prosocial than those who have lower self-transcendence (the organized character) (Cloninger and Zohar, 2011; Josefsson et al., 2011; Cloninger, 2013; Schütz et al., 2013; Zwir et al., 2020a; Moreira et al., 2023a). Studies have shown that adolescents with a creative character are more engaged in school (Moreira et al., 2021b) and more likely to adopt a deep approach to learning (Moreira and Dias, 2019; Moreira and Lee, 2020; Moreira et al., 2021c); and that adults with this character are more likely to have a ‘light’ sense of humor (including benevolent humor; Moreira et al., 2022a), and be the least emotionally reactive (Moreira et al., 2022b).

### 1.3. Integrated temperament-character networks

An important prediction of Cloninger’s personality model is that through principles of equifinality and multifinality, it is theoretically plausible for a person to have any pairing of temperament profile and character profile. While temperament does constrain character development, a person’s temperament profile does not fully determine character development due to the influence of social learning and the stochastic effects of experience (Cloninger et al., 1997), meaning there is no one-to-one correspondence between temperament and character profiles. The occurrence of all combinations of temperament and character profiles is well supported (Cloninger, 2004; Garcia et al., 2019; Moreira et al., 2022c,d), although the likelihood of each combination differs on average. For example, in a sample of adults from the U.S.A, individuals with an explosive temperament profile were most likely to have an apathetic (44%) or disorganized character profile (35%), although 7% had the healthiest creative character. In contrast, people with a reliable temperament profile were most likely to have a creative (38%) or organized character profile (21%), although 6% had the least healthy apathetic character (Cloninger et al., 1997).

Recently, behavioral-genetic studies found that people can be classified into three nearly disjoint networks of temperament and character profiles, each strongly correlated with distinct sets of genes, *which account for these patterns of associations between temperament profiles and character profiles* (Zwir et al., 2021). Each integrated temperament-character network most prominently expresses the prototypical features of one the three major system of human learning and memory that evolved sequentially across human evolution: associative conditioning, intentionality, and self-awareness (Zwir et al., 2022; Garcia et al., 2023).

People in the emotional-unreliable network, who prominently express the system for associative conditioning, typically have a temperament profile featuring high novelty seeking and/or high harm avoidance and a character profile featuring low self-directedness and cooperativeness. Consequently, such individuals are expected to be emotionally reactive, driven by fear, desire and habits, and are generally unhealthy and susceptible to magical thinking and unrealistic social messaging (Garcia et al., 2023; Zwir et al., 2023). Consistent with this, studies classifying participants into the three integrated temperament-character networks have found those in the emotional-unreliable network have lower than average engagement in school (Moreira et al., 2021b), a ‘dark’ sense of humor featuring high sarcasm and cynicism comic styles (Moreira et al., 2022a), higher than average psychological reactance (Moreira et al., 2022b), elevated negative affect and lower life satisfaction (Moreira et al., 2023a), and a higher probability of ill-being (Zwir et al., 2021).

People in the organized-reliable network, who most prominently express the system for intentionality, are more likely to have a reliable temperament with high persistence and are distinguished by an organized character profile. Given these psychobiological features, these people are tolerant and helpful and have a tendency to be capable of resourceful productivity, but are also conventional, materialistic and primarily concerned with their own interests or those of close friends/associates (Garcia et al., 2023). Such features are typical of successful people in Western societies (Cloninger, 2013).

Finally, people in the creative-reliable network, who most prominently express the most recently evolving human system of human learning and memory – that of self-awareness – have a reliable temperament combined with a creative character profile. These people are expected to be capable of resourceful productivity and helpful cooperation like those in the organized-reliable network, but also to be more intuitive, meditative and creative. Research has shown that these individuals experience the highest levels of positive affect and wellbeing (Zwir et al., 2021; Garcia et al., 2022; Moreira et al., 2023a), are the most virtuous (Moreira et al., 2022c), have the lightest sense of humor (including styles of humor that aim at the good; Moreira et al., 2022a), and have the healthiest subjective experiences of their environments (e.g., engagement with school; Moreira et al., 2021b).

### 1.4. Person-centered approach in psycholexical models

In the last years, there has been a growing interest by person-centered approaches to personality, from the psychobiological model to the psycholexical models. Results from studies aiming at identifying personality profiles in the psycholexical models of personality have been inconsistent. The HEXACO and the Five-Factor models are the most representatives of the psycholexical models of personality. Based on the neuropharmacological evidence that dopamine is associated with exploration and incentive-related action, and serotonin with satiety and constraint, authors such as Hirsh, DeYoung and Peterson suggested that, at broadest level of description, variation in human personality reflects engagement and restraint of behavior, organized in terms of two metatraits: Plasticity (reflecting the shared variance between Extraversion and Openness/Intellect), and the metatrait of Stability (reflecting the shared variance among Neuroticism, Agreeableness, and Conscientiousness). The Plasticity metatrait was theorized to relate to individual differences in the functioning of the dopamine system while the Stability metatrait was theorized to relate to individual differences of the serotonin system (Hirsh et al., 2009). Espinoza et al. (2020), using Latent Profiles Analyses, found that a 5 profiles solution fit their large data well: Profile 1: Achievement-oriented agentic (self-focused achievement orientation); Profile 2 Ego-agentic agentic (agentic orientation focusing on self within a social context); Profile 3: Insecure (high emotionality interfering with task accomplishment and social relationships); Profile 4: Communal (communal rather than task-orientation), and Profile 5: Socially Adjusted (healthy balance of agentic and communal concerns, and emotionally and socially adjusted) (Espinoza et al., 2020). A recent meta-analysis conducted by Yin, Lee, Sheldon, Li and Zhao analysed 34 empirical studies using Big Five model and found that there were four possible personality profile solutions, with the three-profile (Resilient, Undercontrolled and Overcontrolled) and four-profile (Resilient, Undercontroller. Average and Undercontroller) solutions being the more predominant (Yin et al., 2021). The nature of the psycholexical and the psychobiological models is so markedly different that the comparison and discussion of the meaning of the profiles found by the different models would be extremely extensive and clearly fills out the scope of this work.

Although the psycholexical approaches have varied in suggesting from two to five as the number of significant multi-dimensional profiles in the general population, sociological studies have consistently identified three prototypic profiles that are described as traditional, materialists (moderns), and post-materialists (cultural creatives). The most thorough empirical data is available in over 100 countries in the World Values Survey, which represents 90% of the world population (Inglehart, 2018). The characteristics of people in these prototypic configurations correspond closely to those distinguished by their temperament and character configurations: emotional-unregulated people are traditionals whose values depend on authority-dependent conventions and habits), (organized-reliable people are materialists with secular-materialistic goals and egocentric values), and creative-reliable people are cultural creatives with prosocial goals and self-transcendent values) (Cloninger and Zwir, 2022; Garcia et al., 2023). Whether based on cognition, personality, or values, there are consistently three major prototypical groups of people in the general human population who are distinguished by specific patterns of learning and memory, temperament and character, and goals and values.

### 1.5. The present study

As reviewed, current evidence suggests that a complete understanding of human functioning requires consideration of the multi-trait temperament and/or character profiles that describe each person as a whole. Consequently, any researchers and practitioners wanting to describe and understand the personality features of individuals relative to the general population need to consider differences in personality configurations, and not only individual dimensions. Toward this goal, it is essential to have information about the prevalence of different personality profiles and networks within a reference population. As far as we are aware, no study has specifically sought to do this, especially in terms of the recently validated integrated temperament-character networks from the TCI. Moreover, the goal of describing differences (and similarities) between demographically defined groups (e.g., age, gender, education level), including in terms of personality dimensions, is an enduring topic in psychological research that remains relevant for guiding solutions to social issues. However, the new and emerging literature on integrated temperament-character networks has not yet provided a comprehensive description of their distribution across demographic groups.

To address these gaps in knowledge, building on recently published normative data for the European Portuguese TCI-R (Moreira et al., 2023b), the principle aim of the current study was to describe the prevalence of (a) temperament profiles, (b) character profiles, and (c) integrated temperament-character networks within a large representative sample the Portuguese adult population, including a test of the distributions of these higher-order traits as a function of gender, age cohort, and education level.

## 2. Materials and methods

### 2.1. Participants

The participants for this study were the same adults used to estimate normative values for individual temperament and character dimensions for the general continental Portuguese population (Moreira et al., 2023b). The sample comprised 2,443 adults obtained from the Northern, Central and Southern districts of Portugal using a stratified sampling strategy. We used the most recent population census available at the time to determine strata based on geographical location and gender, set a target sample size for each stratum based on its proportion in the general population, and then sampled each stratum using a chain-referral strategy. Twelve participants were excluded for incorrectly responding to one or more of five validity check items incorporated in the TCI-R. Sample characteristics are presented in Table 1. The full distribution of the sample according to age cohort, gender, and education level are shown in Table 2.

**Table 1:** Composition of the study sample compared to the continental Portuguese population (2011 census).

	Portuguese population (*N* = 10,562,178)	Sample (*n* = 2,443)
	% of population	% of sample
Male vs. female	47.7 vs. 52.2%	47.4 vs. 52.5%
Age cohort		
15–19*	5.4	8.1
20–29	11.7	16.6
30–39	15.1	16.4
40–49	14.6	16.0
50–59	13.2	14.7
60–69	11.2	14.2
70+	13.8	14.0
Region		
North	22.7	21.7
Metropolitan Porto	12.2	15.4
Center	22.0	29.7
Metropolitan Lisbon	26.7	22.2
South (Alentejo + Algarve)	11.4	11.1

*The present study recruited individuals aged 17 years +, and thus does not fully cover the 15–19 age range from the 2011 census.

**Table 2:** Distribution of normative Portuguese sample as a function of age cohort, gender, and education level.

	Frequency (%)				Row total
	Primary	Lower secondary	Upper secondary	Tertiary	
17–19					
Female	0 (0)	21 (20)	83 (79)	1 (1)	105
Male	0 (0)	43 (47)	49 (53)	0 (0)	92
20–29					
Female	0 (0)	32 (15)	103 (50)	72 (35)	207
Male	0 (0)	52 (26)	98 (50)	47 (24)	197
30–39					
Female	3 (1)	40 (19)	53 (26)	110 (53)	206
Male	0 (0)	30 (16)	77 (40)	86 (45)	193
40–49					
Female	18 (9)	53 (25)	61 (29)	77 (37)	209
Male	11 (6)	71 (40)	49 (28)	46 (26)	177
50–59					
Female	56 (29)	52 (27)	38 (20)	46 (24)	192
Male	38 (23)	62 (38)	41 (25)	24 (15)	165
60–69					
Female	90 (51)	39 (22)	17 (10)	32 (18)	178
Male	69 (42)	48 (29)	16 (10)	32 (19)	165
70+					
Female	129 (75)	19 (11)	2 (1)	22 (13)	172
Male	114 (71)	34 (21)	5 (3)	7 (4)	160
Column total	528	596	692	602	

### 2.2. Measures

Participants completed the 240-item European Portuguese version of the TCI-R (Moreira et al., 2017), which has seven scales for novelty seeking (35 items), harm avoidance (33 items), reward dependence (30 items), persistence (35 items), self-directedness (40 items), cooperativeness (36 items), and self-transcendence (26 items). All TCI-R items are scored on a 5-point Likert scale from 1 (definitely false) to 5 (definitely true). A psychometric evaluation of the European Portuguese TCI-R dimensions using the same representative sample (Moreira et al., 2023b) showed the seven dimensions had excellent internal consistency reliability (McDonald’s omega range 0.80–0.90).

Participants also responded to items assessing socio-demographic characteristics. Participants reported their age in years and were grouped according to seven age categories. Gender was coded as categorical variable (male vs. female). Finally, from a series of 10 options, participants recorded their highest level of educational attainment. These options were grouped to form four higher-order categories reflecting primary education (maximum attainment of 4th grade or lower), lower secondary education (maximum attainment of 6th grade, 9th grade, or basic vocational training), upper secondary education (maximum attainment of 12th grade), and tertiary education (maximum attainment of degree or post-graduate degree, including Ph.D.).

### 2.3. Profile and network formation

To form temperament profiles, the subjects were divided into groups reflecting those above and below the median for each of the four temperament dimensions. Participants were then grouped according to the 16 possible combinations (e.g., nhrp and NHRP). We label these temperament profiles according to the 3-dimensional profiles shown in Figure 1, but differentiating between profiles with high persistence vs. low persistence (e.g., explosive temperament with low persistence).

To form the character profiles, the subjects were divided into groups reflecting those above and below the median for each of the three character dimensions, and then grouped according to the eight possible combinations (e.g., sct and SCT). We label these character profiles according to the 3-dimensional profiles shown in Figure 2.

**Figure 3:**
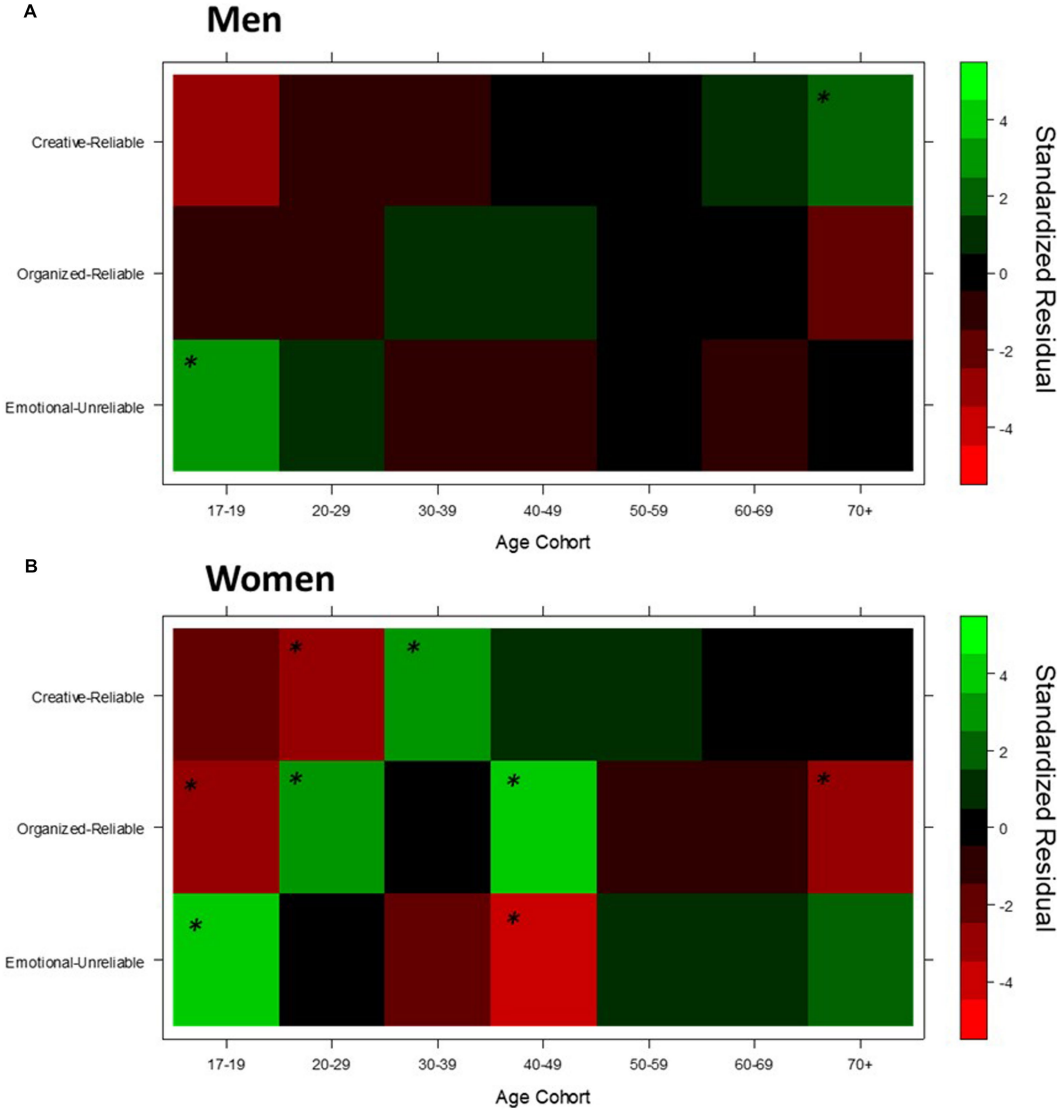
Heat map depicting the association between phenotypic networks and temperament profiles. Colors represent standardized residuals. *z > |1.96|. Panel **(A)** Heat maps of standardized residuals for association between phenotypic networks and temperament profiles in Men. Panel **(B)** Heat maps of standardized residuals for association between phenotypic networks and temperament profiles in Women

Finally, to represent the three integrated temperament-character networks we grouped participants with character profiles featuring low self-directedness (emotional-unreliable network); with character profiles featuring high self-directedness but with low cooperativeness and/or self-transcendence (organized-reliable network); and with a character profile featuring high levels of all three character traits (creative-reliable). Several studies (e.g., Moreira et al., 2021b, 2022a,b, 2023a) have confirmed that these three groupings of character profiles are a valid approximation of the three integrated temperament-character networks identified in behavioral-genetic studies with robust machine-learning algorithms (Zwir et al., 2021) and therefore suitable at capturing the complex patterns of associations between temperament profiles and character profiles that occur in the general population. Confirming this, a chi-squared test indicated that the distribution of temperament profiles across the derived integrated temperament-character networks differed significantly to what would be expected by chance, *χ*^2^ (30) = 537.6, *p* < 0.001. The pattern of standardized residuals for this association aligned with the networks presented by Zwir et al. (2021) and hence validated our method for forming the integrated temperament-character networks (see Figure 3).

### 2.4. Statistical analysis

All analyses were performed using the statistical software R (R Core Team, 2021). First, we described the prevalence of temperament profiles, character profiles, and integrated temperament-character networks in our representative sample.

Next, we explored how gender, age cohort and education level were associated with the personality profiles and networks. For this, we used contingency tables and the chi-squared (*χ*^2^) test. The chi-squared analysis tests whether the frequencies observed in a sample differ from the frequencies that would be expected from a baseline model. Significant chi-squared tests were explored further by examining standardized residuals. Standardized residuals are z-scores, and therefore standardized residuals > |1.96| represent significance at *p* < 0.05 (Field, 2012).

Given our results, we explored how integrated temperament-character networks differ as a function of (a) gender and age cohort, and (b) gender and education level. We tested these interaction effects by performing a log-linear analysis. This included three categorical variables (age cohort or education level, gender, and integrated temperament-character networks) and the full set of interactions between them. This process involves first testing a saturated model with all interaction terms, and then a second model removing the three-way interaction (Field, 2012). A significant change in the likelihood ratio statistic indicates the interaction term is statistically important. When this three-way interaction was significant, we performed additional chi-squared tests on age cohort and integrated temperament-character networks for male and female subjects independently.

## 3. Results

### 3.1. Prevalence of personality profiles in the full sample

Our first aim was to describe the prevalence of temperament profiles, character profiles and integrated temperament-character networks in our normative Portuguese sample (see Tables 3, 7). The distribution of temperament profiles within the sample was significantly different to what would be expected by chance, *χ*^2^ (15) = 442.7, *p* < 0.001. Standardized residuals indicated that the passionate profile with high persistence (12.4% of the sample), methodical profile with low persistence (10.6%), explosive profile with low persistence (8.8%), cautious profile with high persistence (7.5%), and reliable profile with high persistence (7.4%) were more typical (significant types, *z* > 1.96) in the sample. The distribution of character profiles within the sample was also significantly different to what would be expected by chance, χ^2^ (7) = 486.9, *p* < 0.001. Standardized residuals indicated that the apathetic (15.7%), disorganized (18.1%), organized (16.7%) and creative (20.4%) character profiles were more typical in the sample. For the integrated temperament-character networks, we performed a χ^2^ test with the expected frequencies set to reflect the proportion of character profiles included in the network (emotional-unreliable = 50%; organized-reliable = 37.5%; creative-reliable = 12.5%). The analysis indicated a significant departure from this base model, *χ*^2^ (2) = 156.4, *p* < 0.001. Standardized residuals indicated that this significant result was driven by an overrepresentation of the creative-reliable network and an underrepresentation of the organized-reliable network (see Table 4).

**Table 3:** Proportions of different character profiles according to temperament profile.

Temperament Profile	Character Profiles in each temperament profile (%)								Row totals
	Sct “Apathetic”	scT “Disorganized”	sCt “Dependent”	sCT “Moody”	SCt “Bossy”	ScT “Fanatical”	SCt “Organized”	SCT “Creative”	
NHrp “Explosive”	43	30	5	6	9	1	6	1	100
NHrP	16	33	4	18	11	5	9	5	100
Nhrp “Adventurous”	27	24	3	1	21	3	12	8	100
NhrP	15	24	4	1	17	10	15	14	100
NHRp “Sensitive”	19	18	11	19	5	2	16	10	100
NHRP	4	17	6	31	3	7	11	20	100
NhRp “Passionate”	10	10	9	9	12	5	23	23	100
NhRP	3	12	2	7	9	5	20	42	100
nHrp “Methodical”	34	21	10	8	8	4	8	7	100
nHrP	15	28	4	11	4	9	11	18	100
nHRp “Cautious”	11	17	9	15	4	6	16	23	100
nHRP	3	19	7	24	1	3	18	25	100
nhrp “Independent”	18	10	1	4	15	1	32	18	100
nhrP	11	12	2	5	18	8	23	20	100
nhRp “Reliable”	4	4	5	0	2	4	56	25	100
nhRP	3	5	3	5	3	6	25	49	100

Values in bold and paired with an asterisk reflect z > 1.96. Values paired with ^†^ reflect *z* > −1.96.

**Table 4:** Distribution of temperament profiles, character profiles and temperament-character networks by gender.

	Full sample	Frequency (%)	
		♂	♀
Temperament profile			
NHrp “Explosive”	216 (8.8)	99 (8.5)	117 (9.1)
NHrP	57 (2.3)	24 (2.1)	33 (2.6)
Nhrp “Adventurous”	147 (6.0)	100 (8.6)^*^	47 (3.7)^†^
NhrP	156 (6.4)	102 (8.8)^*^	54 (4.2)^†^
NHRp “Sensitive”	153 (6.3)	46 (4.0)^†^	107 (8.3)*
NHRP	122 (5.0)	32 (2.8)^†^	89 (6.9)*
NhRp “Passionate”	128 (5.2)	57 (4.9)	71 (5.5)
NhRP	303 (12.4)	134 (11.6)	169 (13.2)
nHrp “Methodical”	258 (10.6)	122 (10.5)	136 (10.6)
nHrP	134 (5.5)	57 (4.9)	77 (6.0)
nHRp “Cautious”	149 (6.1)	49 (4.2)^†^	100 (7.8)^*^
nHRP	182 (7.5)	65 (5.6)^†^	117 (9.1)^*^
nhrp “Independent”	68 (2.8)	46 (4.0)^*^	22 (1.7)^†^
nhrP	133 (5.4)	91 (7.9)^*^	42 (3.3)^†^
nhRp “Reliable”	55 (2.3)	32 (2.8)	23 (1.8)
nhRP	181 (7.4)	102 (8.8)	79 (6.2)
Character profile			
sct “Apathetic”	384 (15.7)	220 (19.0)^*^	164 (12.8)^†^
scT “Disorganized”	441 (18.1)	217 (18.7)	223 (17.4)
sCt “Dependent”	134 (5.5)	56 (4.8)	78 (6.1)
sCT “Moody”	247 (10.1)	73 (6.3)^†^	174 (13.6)^*^
Sct “Bossy”	211 (8.6)	126 (10.9)^*^	85 (6.6)^†^
ScT “Fanatical”	119 (4.9)	62 (5.3)	57 (4.4)
SCt “Organized”	408 (16.7)	203 (17.5)	205 (16.0)
SCT “Creative”	498 (20.4)	201 (17.3)^†^	297 (23.1)^*^
Temperament-Character network			
Emotional-Unreliable	1,206 (49.4)	566 (48.8)	639 (49.8)
Organized-Reliable	738 (30.2)	391 (33.7)^*^	347 (27.0)^†^
Creative-Reliable	498 (20.4)	201 (17.3)^†^	297 (23.1)^*^

Values in bold and paired with an asterisk reflect z > 1.96. Values paired with ^†^ reflect *z* > −1.96.

### 3.2. Prevalence of personality profiles as a function of gender

There was a significant association between gender and temperament profile, *χ*^2^ (15) = 154.5, p < 0.001. Based on standardized residuals, it was evident that this association was driven by a greater prevalence of independent, reliable (high persistence only), and adventurous temperament profiles in men, and a greater prevalence of cautious and sensitive temperament profiles in women. A chi-squared test also showed a significant association between gender and character profile, *χ*^2^ (7) = 73.65, *p* < 0.001. Standardized residuals showed that this association was driven by a greater prevalence of apathetic and bossy profiles in men, and a greater prevalence of moody and creative profiles in women. Finally, we found a significant association between gender and integrated temperament-character networks, *χ*^2^ (2) = 19.20, p < 0.001. This significant effect was driven by a higher prevalence of men than expected in the organized-reliable network and a higher prevalence of women than expected in the creative-reliable network.

### 3.3. Prevalence of personality profiles as a function of age cohort

A chi-squared test revealed a significant association between age group and temperament profile, *χ*^2^ (90) = 328.49, *p* < 0.001. Standardized residuals showed temperament profiles including high novelty-seeking were prevalent in younger adults whereas temperament profiles including low novelty-seeking were prevalent in older adults (see Table 5). It was also noteworthy that temperament profiles with high harm avoidance were typical of adults aged 70+. There was a significant association between age cohort and character profile, *χ*^2^ (42) = 117.9, *p* < 0.001. From the standardized residuals, it was clear the association between age and character profile was complex. However, some patterns were discernable: (a) the apathetic profile was typical of the youngest cohort; (b) the dependent profile was typical of adults in their 20s; (c) the organized profile was typical of adults aged 30–49; (d) the distribution of character profiles did not differ significantly from chance for adults aged 50–69; and (e) character profiles with high self-transcendence were typical of adults aged 70+. Finally, there was a significant association between age cohort and network, χ^2^(12) = 58.77, p < 0.001. Based on standardized residuals, it was apparent that younger adults typically occupied the emotional-unreliable network, that middle-aged adults typically occupied the organized-reliable network, and that older adults (50+ years) typically occupied the creative-reliable.

**Table 5:** Distribution of temperament profiles, character profiles and temperament-character networks by age cohort.

	Frequency (%)						
	17–19	20–29	30–39	40–49	50–59	60–69	70+
Temperament profile							
NHrp “Explosive”	15 (7.6)	32 (7.9)	37 (9.2)	35 (9.0)	39 (10.8)	25 (7.2)	33 (9.7)
NHrP	5 (2.5)	13 (3.2)	12 (3.0)	9 (2.3)	4 (1.1)	9 (2.6)	5 (1.5)
Nhrp “Adventurous”	20 (10.1)^*^	25 (6.2)	21 (5.2)	28 (7.2)	18 (5.0)	21 (6.1)	14 (4.1)
NhrP	16 (8.1)	29 (7.2)	34 (8.5)	25 (6.4)	23 (6.4)	18 (5.2)	11 (3.2)^†^
NHRp “Sensitive”	21 (10.6)^*^	40 (9.9)^*^	26 (6.5)	25 (6.4)	17 (4.7)	12 (3.5)^†^	12 (3.5)^†^
NHRP	12 (6.1)	28 (6.9)	27 (6.7)	17 (4.4)	15 (4.2)	13 (3.7)	10 (2.9)
NhRp “Passionate”	18 (9.1)^*^	24 (5.9)	24 (6.0)	22 (5.6)	16 (4.4)	16 (4.6)	8 (2.3)^†^
NhRP	34 (17.2)	82 (20.2)^*^	74 (18.5)^*^	46 (11.8)	30 (8.3)^†^	27 (7.8)^†^	10 (2.9)^†^
nHrp “Methodical”	19 (9.6)	21 (5.2)^†^	26 (6.5)^†^	32 (8.2)	50 (13.9)	51 (14.7)^*^	59 (17.3)^*^
nHrP	8 (4.0)	13 (3.2)	14 (3.5)	21 (5.4)	25 (6.9)	19 (5.5)	34 (10.0)^*^
nHRp “Cautious”	5 (2.5)^†^	14 (3.5)^†^	19 (4.7)	16 (4.1)	23 (6.4)	31 (8.9)^*^	41 (12.0)^*^
nHRP	9 (4.5)	19 (4.7)^†^	17 (4.2)^†^	31 (7.9)	24 (6.7)	38 (11.0)^*^	44 (12.9)^*^
nhrp “Independent”	4 (2.0)	10 (2.5)	8 (2.0)	18 (4.6)^*^	13 (3.6)	12 (3.5)	3 (0.9)^†^
nhrP	3 (1.5)^†^	27 (6.7)	26 (6.5)	22 (5.6)	19 (5.3)	16 (4.6)	20 (5.9)
nhRp “Reliable”	1 (0.5)	5 (1.2)	5 (1.2)	13 (3.3)	10 (2.8)	16 (4.6)^*^	5 (1.5)
nhRP	8 (4.0)	23(5.7)	31 (7.7)	30(7.7)	34 (9.4)	23 (6.6)	32 (9.4)
Character profile							
sct “Apathetic”	49 (24.7)^*^	63 (15.6)	57 (14.2)	55 (14.1)	65 (18.1)	49 (14.1)	46 (13.5)
scT “Disorganized”	43 (21.7)	70 (17.3)	66 (16.5)	48 (12.3)^†^	71 (19.7)	72 (20.7)	71 (20.8)
sCt “Dependent”	14 (7.1)	32 (7.9)^*^	23 (5.7)	24 (6.2)	13 (3.6)	15 (4.3)	13 (3.8)
sCT “Moody”	25 (12.6)	42 (10.4)	31 (7.7)	30 (7.7)	32 (8.9)	35 (10.1)	52 (15.2)^*^
Sct “Bossy”	15 (7.6)	39 (9.6)	36 (9.0)	44 (11.3)	34 (9.4)	27 (7.8)	16 (4.7)^†^
ScT “Fanatical”	8 (4.0)	18 (4.4)	13 (3.2)	18 (4.6)	19 (5.3)	15 (4.3)	28 (8.2)^*^
SCt “Organized”	21 (10.6)^†^	79 (19.5)	83 (20.7)	85 (21.8)^*^	49 (13.6)	56 (16.1)	35 (10.3)^†^
SCT “Creative”	23 (11.6)^†^	62 (15.3)^†^	92 (22.9)	86 (22.1)	77 (21.4)	78 (22.5)	80 (23.5)
Temperament-Character network							
Emotional-Unreliable	131 (66.2)^*^	207 (51.1)	177 (44.1)	157 (40.3)^†^	181 (50.3)	171 (49.3)	182 (53.4)
Organized-Reliable	44 (22.2)^†^	136 (33.6)	123 (32.9)	147 (37.7)^*^	102 (28.3)	98 (28.2)	79 (23.2)^†^
Creative-Reliable	23 (11.6)^†^	62 (15.3)^†^	92 (22.9)	86 (22.1)	88 (21.4)	78 (22.5)	80 (23.5)

Values in bold and paired with an asterisk reflect z > 1.96. Values paired with ^†^ reflect z > −1.96

### 3.4. Associations between personality and education level

The analysis revealed a significant association between temperament profile and education level, χ^2^ (75) = 257.79, p < 0.001. Standardized residuals showed that the cautious temperaments were typical of participants with 4th grade education or less (Table 6). In contrast, the passionate temperament (high persistence only) was typical of those with the higher levels of education. There was also a significant association between character profile and education level, χ^2^ (35) = 128.04, *p* < 0.001. The standardized residuals from this analysis showed that the creative and organized character profiles were typical of participants with a degree. In contrast, the apathetic character profile was typical of those with a maximum attainment of the 6th or 9th grade. The disorganized and fanatical character profiles were typical of those with a maximum attainment of the 4th grade. Finally, while not significant, adults with the lowest educational attainment were linked to the dependent and moody profiles. Finally, there was a significant association between integrated temperament-character network and education level, χ^2^ (10) = 48.55, p < 0.001. Based on standardized residuals, it was evident that the organized-reliable and creative-reliable networks were typical of participants with a degree. While not significant at *p* < 0.05, there was a trend for the emotional-unreliable network to be typical of participants with a maximum education attainment of 9th grade or less.

**Table 6:** Distribution of temperament profiles, character profiles and integrated temperament-character networks by education level.

	Frequency (%)			
	Primary	Lower secondary	Upper secondary	Tertiary
Temperament profile				
NHrp “Explosive”	49 (9.3)	69 (11.6)^*^	60 (8.7)	32 (5.3)^†^
NHrP	8 (1.5)	15 (2.5)	23 (3.3)	11 (1.8)
Nhrp “Adventurous”	27 (5.1)	38 (6.4)	44 (6.3)	36 (6.0)
NhrP	22 (4.2)^†^	33 (5.5)	59 (8.5)^*^	41 (6.8)
NHRp “Sensitive”	19 (3.6)^†^	37 (6.2)	53 (7.6)	43 (7.1)
NHRP	15 (2.8)^†^	29 (4.9)	42 (6.1)	35 (5.8)
NhRp “Passionate”	16 (3.0)^†^	27 (4.5)	43 (6.2)	41 (6.8)
NhRP	27 (5.1)^†^	60 (10.1)	107 (15.4)^*^	108 (17.9)^*^
nHrp “Methodical”	94 (17.8)^*^	58 (9.7)	56 (8.1)^†^	49 (8.1)
nHrP	53 (10.0)^*^	25 (4.2)	28 (4.0)	26 (4.3)
nHRp “Cautious”	52 (9.8)^*^	32 (5.4)	32 (4.6)	33 (5.5)
nHRP	59 (11.2)^*^	53 (8.9)	33 (4.8)^†^	36 (6.0)
nhrp “Independent”	10 (1.9)	27 (4.5)^*^	17 (2.5)	14 (2.3)
nhrP	30 (5.7)	34 (5.7)	37 (5.3)	29 (4.8)
nhRp “Reliable”	10 (1.9)	14 (2.3)	13 (1.9)	17 (2.8)
nhRP	37 (7.0)	45 (7.6)	46 (6.6)	51 (8.5)
Character profile				
sct “Apathetic”	81 (15.3)	127 (21.3)^*^	113 (16.3)	59 (9.8)^†^
scT “Disorganized”	129 (24.4)^*^	108 (18.1)	122 (17.6)	75 (12.5)^†^
sCt “Dependent”	20 (3.8)	32 (5.4)	40 (5.8)	42 (7.0)
sCT “Moody”	57 (10.8)	61 (10.2)	66 (9.5)	57 (9.5)
Sct “Bossy”	46 (8.7)	44 (7.4)	74 (10.7)	46 (7.6)
ScT “Fanatical”	37 (7.0)^*^	31 (5.2)	25 (3.6)	25 (4.2)
SCt “Organized”	62 (11.7)^†^	87 (14.6)	117 (16.9)	141 (23.4)^*^
SCT “Creative”	96 (18.2)^†^	106 (17.8)	136 (19.6)	157 (26.1)^*^
Temperament-Character network				
Emotional-Unreliable	287 (54.4)	328 (55.0)^*^	341 (49.2)	233 (38.7)^†^
Organized-Reliable	145 (27.5)	162 (27.2)	216 (31.2)	212 (35.2)^*^
Creative-Reliable	96 (18.2)	106 (17.8)	136 (19.6)	157 (26.1)^*^

Values in bold and paired with an asterisk reflect z > 1.96. Values paired with ^†^ reflect *z* > −1.96.

### 3.5. Interaction effects: age cohort and education level effects as a function of gender

**Figure 4:**
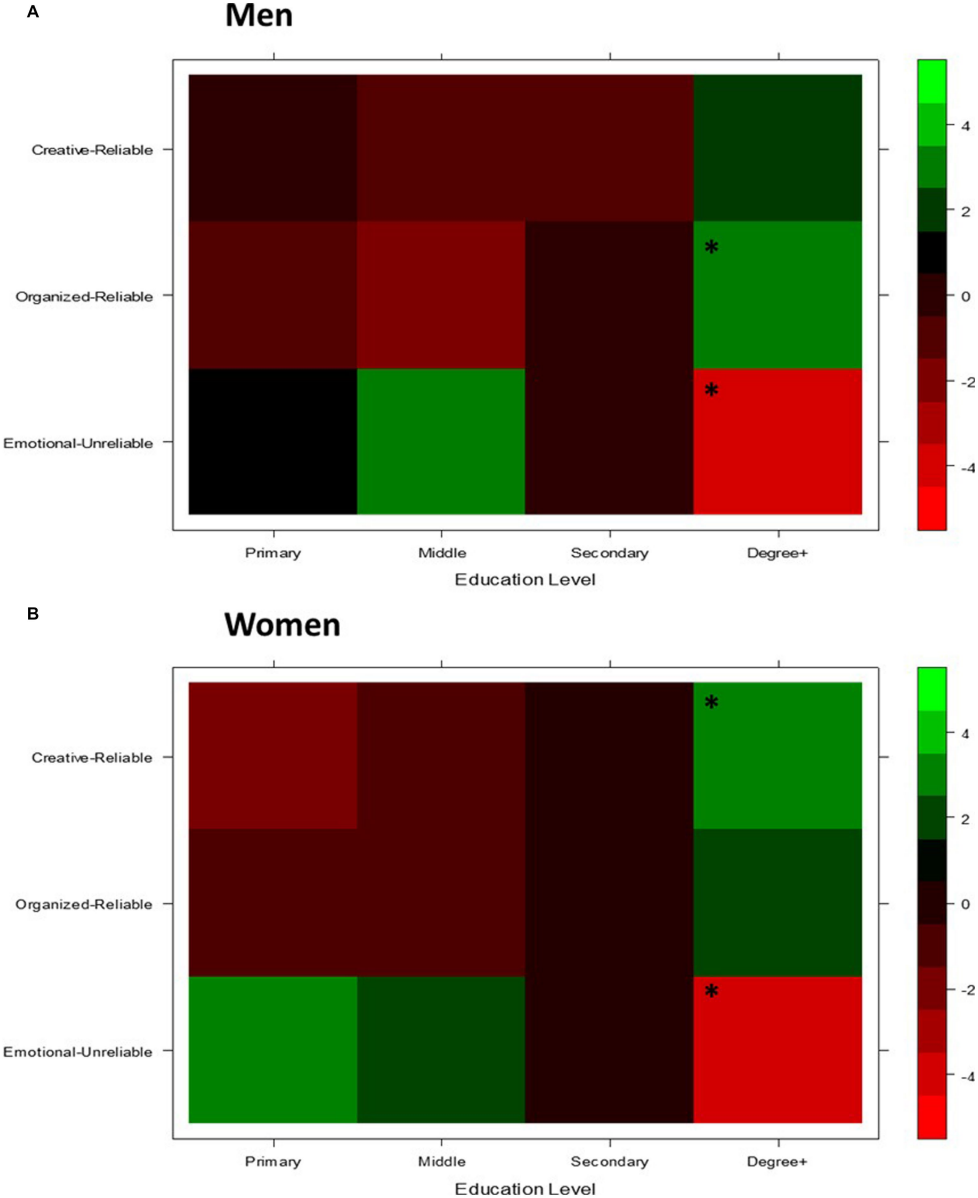
Heat maps of standardized residuals for association between integrated temperament-character networks and age cohort for men and women separately. Green represents positive values (occurs more frequently than expected). Red represents negative values (occurs less frequently than expected). *Standardized residuals > |1.96|, correspond to *p* < 0.05. Panel **(A)** Heat maps of standardized residuals for association between integrated temperament-charcater networks and age in Man. Panel **(B)** Heat maps of standardized residuals for association between integrated temperament-charcater networks and age in Women.

Age cohort × gender. The three-way log-linear analysis retained all effects, meaning the three-way age cohort × gender × integrated temperament-character network interaction was statistically significant, χ^2^ (12) =22.82, *p* = 0.029. Follow-up chi-squared tests showed there was a significant association between age cohort and network for women, χ^2^ (12) =61.03, p < 0.001, but not for men, χ^2^ (12) =20.02, *p* = 0.067 (Figure 4).

**Figure 5:**
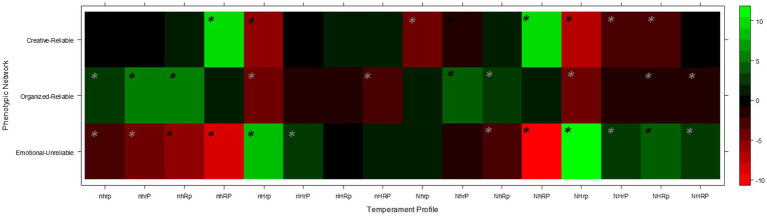
Heat maps of standardized residuals for association between integrated temperament-character networks and education level for men and women separately. Green represents positive values (occurs more frequently than expected). Red represents negative values (occurs less frequently than expected). *Standardized residuals > |1.96|, correspond to *p* < 0.05.

Education level × gender. The three-way log-linear analysis did not retain all effects, meaning the three-way education level × gender × integrated temperament-character network interaction was not statistically significant, χ^2^ (6) =3.81, *p* = 0.703 (Figure 5). Separate chi-squared tests showed there were significant associations between education level and network for both men, *χ*^2^(6) =20.75, *p* = 0.002, and women, *χ*^2^(6) =24.74, *p* < 0.001.

## 4. Discussion

Complementing the normative values for individual temperament and character dimensions presented elsewhere (Moreira et al., 2023b) the present study offers a comprehensive description of the multi-trait personality profiles and networks as they occur within the continental Portuguese population (see Table 7). Specifically, we presented normative frequencies for temperament profiles, character profiles, and integrated temperament-character networks for the whole representative sample as well as separately for men vs. women, across multiple age cohorts ranging from youth (17–19 years) to old age (70+ years), and across adults with different educational attainment. We consider each of these results in turn:

**Table 7:** Full frequency table of temperament profiles, character profiles, and joint temperament-character networks.

Network	Character Profile	Temperament Profile																C Total	N Total
		Independent		Reliable		Methodical		Cautious		Adventurous		Passionate		Explosive		Sensitive			
		nhrp	nhrP	nhRp	nhRP	nHrp	nHrP	nHRp	nHRP	Nhrp	NhrP	NhRp	NhRP	NHrp	NHrP	NHRp	NHRP		
Emo-Un.	sct	12	14	2	6	88	20	16	5	40	23	13	10	92	9	29	5	384	1,206
	scT	7	16	2	9	54	38	25	35	36	37	13	37	64	19	28	21	441	
	sCt	1	3	3	6	26	5	13	13	5	6	11	5	11	2	17	7	134	
	sCT	3	7	0	9	20	15	22	44	2	2	11	22	13	10	29	38	247	
Org-Rel.	Sct	10	24	1	6	21	5	6	2	31	27	15	27	19	6	7	4	211	738
	ScT	1	11	2	11	11	12	9	5	4	16	6	15	2	3	3	8	119	
	SCt	22	31	31	46	20	15	24	33	17	23	29	61	13	5	24	14	408	
Cre-Rel.	SCT	12	27	14	88	18	24	34	45	12	22	30	126	2	3	16	25	498	498
	T Total	68	133	55	181	258	134	149	182	147	156	128	303	216	57	153	122		

Emo-Un, Emotional-Unreliable Network; Org-Rel, Organized-Reliable Network; Cre-Rel, Creative-Reliable Network.

A large body of research has demonstrated the nonlinear dynamics of personality development via the principals of equifinality and multifinality (Cloninger et al., 1997; Cloninger, 2004). All combinations of temperament and character are theoretically plausible (but not necessarily as likely), with the same character profile having different antecedent temperament profiles (equifinality) and the same temperament profile having different outcomes in terms of character coherence (multifinality). Aligning with these principals, our study showed that all but one of the 128 possible combinations of temperament profiles and character profiles emerged in the sample. Moreover, we found that certain profiles were more prevalent than others. For example, aligning with prior works (Cloninger et al., 1997; Cloninger, 2009), we found that individuals with an explosive temperament were far more likely to have an immature apathetic character than a mature creative character. Also consistent with past studies, we found that individuals with a reliable temperament (nhR) with high persistence were particularly predisposed to have a well-developed character.

### 4.1. Gender differences

When assessing differences between men and women in the Portuguese population we found several significant differences. This is noteworthy given that almost all differences between men and women for individual TCI traits were of a negligible effect size (Moreira et al., 2023b). All temperament configurations were evident in both sexes, but we found profiles with a combination of low harm avoidance and low reward dependence were typical (more cases than expected under the base model) of the men while profiles with a combination of high harm avoidance and high reward dependence were typical of women. All character configurations were also evident in both sexes. However, the most severely immature character profile was identified as a type in men while the healthiest creative character profile was typical in women. Finally, we found that the organized-reliable network was typical of men, implying that on average, men displayed traits of being capable of resourceful productivity but more conventional, materialistic and practical, and not always fully cooperative with others. In turn, the creative-reliable phenotypic network was shown to be typical of women, implying that the average Portuguese women was more likely to be capable of resourceful productivity and helpful cooperation combined with being more intuitive, meditative and creative.

### 4.2. Differences between age cohorts

The study revealed several differences in personality between the age cohorts. Specifically, we found that a small number of temperament profiles were typical of certain age cohorts. First, temperament profiles characterized by the combination of low novelty seeking and high harm avoidance were typical of older retirement-aged adults, as has been identified in other cross-sectional studies focused on individual traits (Trouillet and Gana, 2008; Calvet et al., 2016). Individuals with this combination of traits usually show behavior that is patient, quiet and serenity seeking (Cloninger, 1987). Second, we found that three profiles with the shared configuration of high novelty seeking (more eager to explore new ways to do things) and low persistence (quick to abandon previously rewarded behavior) were typical of the youngest cohort. This implies that individuals in their late teens differ from other adults in that they still show signs of the developmental changes typically observed in adolescents pertaining to identity formation and emancipation from adult authority (Zohar et al., 2019).

We also found that the most immature character profile (the apathetic character) was typical of the youngest adults while the organized profile -- which is linked to being highly self-confident, responsible, resourceful, tolerant and helpful, but also materialistic and practical rather than meditative and spiritual (Cloninger, 2013) -- was typical of adults in their 40’s. Indeed, the organized character profile was found to be an ‘antitype’ of the very youngest and very oldest age cohorts, implying a nonlinear effect with age. Although not statistically significant, it was apparent that the oldest age cohort had the largest proportion of creative-reliable network (23.5%). These results are consistent with the idea that mean levels of personality traits can be shaped by socialization effects. For example, adults in their 40s are likely to be strongly invested in their careers (and more likely than younger adults to be at a stage when they are starting senior positions) and, therefore, are likely to experience strong social pressures to work hard and get along with colleagues; that is, to be self-directed and cooperative. Having this type of mature character is advantageous because it allows an individual to regulate their emotions responsibly and behave rationally, which in turn helps one to work effectively and engage in culturally-valued tasks. Our finding that Portuguese adults in their 40s typically presented an organized character profile is consistent with observed developmental trends identified in a longitudinal study of Finnish adults (Josefsson et al., 2013). The fact that the oldest Portuguese adults had the highest prevalence of character profiles with high self-transcendence is also consistent with prior cross-sectional studies (Cloninger, 2003). One reason for this elevated self-transcendence is that older people will start to face more death and confront their mortality, for which high self-transcendence is adaptive (Cloninger, 2004).

### 4.3. Differences in personality as a function of education

It is important to acknowledge that our data were cross-sectional, meaning that cohort effects may be critical for understanding the observed effect of age cohort. One such generational difference is maximum educational attainment, which is known to vary markedly across age groups in Portugal (owing to factors such as the dictatorship in the first half of the 20th century). Consistent with this, we observed that retirement age adults in our sample were far more likely to have a primary or lower secondary level of education compared to working-aged adults (see Table 2).

When assessing personality differences as a function of education we found that the highest level of character maturity and personality coherence (including high self-transcendence alongside self-directedness and cooperativeness) was linked to a tertiary education. In turn, we found that immature character and unregulated emotional reactivity was linked to the lowest levels of educational attainment. This effect was broadly the same for men and women. As far as we are aware, no other prior study has systematically shown how TCI traits or profiles vary as a function of educational attainment. However, our findings align with those of studies using alternative personality models. This includes the observed positive correlation between years of education and lexically-derived Conscious Restraint and Openness/Intellect personality dimensions (Goldberg et al., 1998). Conscious restraint reflects traits such as being self-disciplined, socially responsible, and controlled, and is thus conceptually proximal to self-directedness and the notion of intentional and rational self-regulation. In turn, Openness/Intellect captures individuals who are imaginative, innovative, perceptive, philosophical, and interested in truth and beauty, and therefore shares partial conceptual overlap with self-transcendence (as has been supported empirically; De Fruyt et al., 2000).

The capacity to be self-directed and cooperative with others is not only strongly encouraged in education settings (Moreira et al., 2010), but also highly advantageous to being successful during academic trajectories, including Higher education. Thus, norms, values and practices around higher education are all likely to exert an important influence on the development of a mature character (Caspi et al., 2005). However, our results show that people with the highest level of education were not only more mature in an organized sense (self-directed and cooperative), but also in a creative self-aware sense (with the addition of high self-transcendence). Creativity refers to original, adaptive and beneficial innovation linked to a creative state of mind characterized by “calm alertness with a flowing intuitive awareness that awakens automatic intelligences” (p7. Cloninger and Cloninger, 2013). One possibility is that personality change toward creativity can occur in the context of tertiary education because high intellectual and attentional demands leads to distinct patterns of neural activation. For example, neuroscientific studies have shown that sustained attention is associated with high activation of the prefrontal cortex, which can in turn lead to deafferentation of the parietal lobe. Such cessation of neural signaling to this part of the brain has been linked to feelings of unit with one’s environment, which is a key feature of self-transcendence (Newberg and D’Aquili, 2000).

### 4.4. Interaction effects

A noteworthy finding in this study was the observed age cohort-by-gender interaction effect on integrated temperament-character network. Specifically, while there was a general trend for older men to have the more integrated and coherent personalities, personality coherence appeared to be greatest in women in their 30s. Because personality coherence was highest in adults with the best educational attainments, and because middle-aged women were more likely to have a tertiary education than elderly women and late teens, it appears that age cohort had relatively little influence on personality for women. Put differently, our finding strongly implied that education level was a stronger predictor of personality coherence in women than decade of birth. This leaves the question of why the same effect was not observed for men, especially as men showed similar patterns of educational attainment across age cohorts as women.

### 4.5. Limitations

This study has some limitations that are important to consider. Firstly, the TCI-R is a self-report instrument, meaning that some individuals may have been inclined to present themselves favorably rather than truthfully. However, the TCI-R has been validated extensively, and so we expect our results to be reproducible. Second, we note that this study considered the three self-transcendence dimensions included in the original TCI. Updates to the TCI (Cloninger and Zwir, 2022) since our data collection now posit five dimensions. Thus, researchers may in the future wish to update the normative data presented here to include the new ST dimensions.

## 5. Conclusion

This study is the first to characterize a normative sample in terms of its temperament profiles, character profiles, and integrated temperament-character networks. All combinations of TCI traits are theoretically possible, although the study demonstrates that certain configurations were more prevalent among Portuguese adults than others. Moreover, we demonstrate that the prevalence of profiles and networks varied as a function of age cohort, gender, and education level. Independent of age and education, women were more likely to be capable of resourceful productivity and helpful cooperation combined with being more intuitive, meditative and creative than men. Independent of age and gender, individuals with a degree were also more likely to present these biopsychosocial features. We also found that the organized character profile (typical of leaders and successful individuals in Western cultures, linked to being highly self-confident, responsible, resourceful, tolerant and helpful, but also materialistic and practical rather than meditative and spiritual; Cloninger, 2013) was most typical of adults in their 40s. Finally, the distribution of personality profiles across age differed as a function of gender: for men the oldest individuals had the most coherent personalities while high personality integration was most prevalent for women in their 30s.

## Data availability statement

The datasets presented in this article are not readily available because the data is copyrighted. Requests to access the datasets should be directed to pmoreira@utad.pt.

## Ethics statement

The studies involving human participants were reviewed and approved by CIPD. The patients/participants provided their written informed consent to participate in this study.

## Author contributions

PM conceptualization, project administration, resources, investigation, methodology, supervision, writing – original draft preparation, and review and editing. RI data curation, formal analysis, software, visualization, and writing – original draft preparation. RC conceptualization and writing – review and editing. All authors contributed to the article and approved the submitted version.

## Funding

This work was supported by Portuguese Funds through the Portuguese Foundation for Science and Technology (FCT), under the multi-year funding awarded to CIPD [grant number UIDB/04375/2020].

## Conflict of interest

The authors declare that the research was conducted in the absence of any commercial or financial relationships that could be construed as a potential conflict of interest.

## Publisher’s note

All claims expressed in this article are solely those of the authors and do not necessarily represent those of their affiliated organizations, or those of the publisher, the editors and the reviewers. Any product that may be evaluated in this article, or claim that may be made by its manufacturer, is not guaranteed or endorsed by the publisher.
