# Supplementary material for: Three joint temperament-character configurations account for learning, personality and well-being: normative demographic findings in a representative national population
Source: Front Psychol. 2023 Jul 3;14:1193441. doi: 10.3389/fpsyg.2023.1193441 (PMC10393030; doi:10.3389/fpsyg.2023.1193441)
Supplement: Supplementary file 1 [file Table_1.DOCX]

**Supplementary Materials**

ST1. Observed frequencies, expected frequencies, and standardized residuals (*z*) for the relationship between temperament profile and age cohort. Full Sample.

ST2. Observed frequencies, expected frequencies, and standardized residuals (*z*) for the relationship between temperament profile and age cohort. Men

ST3. Observed frequencies, expected frequencies, and standardized residuals (*z*) for the relationship between temperament profile and age cohort. Women

ST4. Observed frequencies, expected frequencies, and standardized residuals (*z*) for the relationship between character profile and age cohort. Full Sample.

ST5. Observed frequencies, expected frequencies, and standardized residuals (*z*) for the relationship between character profile and age cohort. Men

ST6. Observed frequencies, expected frequencies, and standardized residuals (*z*) for the relationship between character profile and age cohort. Women

ST7. Observed frequencies, expected frequencies, and standardized residuals (*z*) for the relationship between phenotypic network and age cohort. Full Sample.

ST8. Observed frequencies, expected frequencies, and standardized residuals (*z*) for the relationship between phenotypic network and age cohort. Men

ST9. Observed frequencies, expected frequencies, and standardized residuals (*z*) for the relationship between phenotypic network and age cohort. Women

ST10. Observed frequencies, expected frequencies, and standardized residuals (*z*) for the relationship between temperament profile and gender

ST11. Observed frequencies, expected frequencies, and standardized residuals (*z*) for the relationship between character profile and gender

ST12. Observed frequencies, expected frequencies, and standardized residuals (*z*) for the relationship between phenotypic network and gender

ST13. Distribution of achieved education level for each age cohort

ST14. Full frequency table of temperament profiles, character profiles, and joint temperament-character networks.

| ST1. Observed frequencies, expected frequencies, and standardized residuals (*z*) for the relationship between temperament profile and age cohort  Full sample | | | | | | | | | | | | | | | | |
| --- | --- | --- | --- | --- | --- | --- | --- | --- | --- | --- | --- | --- | --- | --- | --- | --- |
|  | Independent | | Reliable | | Methodical | | Cautious | | Adventurous | | Passionate | | Explosive | | Sensitive | |
|  | nhrp | nhrP | nhRp | nhRP | nHrp | nHrP | nHRp | nHRP | Nhrp | NhrP | NhRp | NhRP | NHrp | NHrP | NHRp | NHRP |
| 17-19 |  |  |  |  |  |  |  |  |  |  |  |  |  |  |  |  |
| Observed freq. | 4 | 3 | 1 | 8 | 19 | 8 | 5 | 9 | 20 | 16 | 18 | 34 | 15 | 5 | 21 | 12 |
| Expected freq. | 5.5 | 10.8 | 4.5 | 14.7 | 20.9 | 10.9 | 12.1 | 14.8 | 11.9 | 12.6 | 10.4 | 24.6 | 17.5 | 4.6 | 12.4 | 9.9 |
| *z* | -0.7 | -2.5^*^ | -1.7 | -1.9 | -0.5 | -0.9 | -2.2^*^ | -1.6 | 2.5^*^ | 1.0 | 2.5^*^ | 2.1^*^ | -0.7 | 0.2 | 2.6^**^ | 0.7 |
| 20-29 |  |  |  |  |  |  |  |  |  |  |  |  |  |  |  |  |
| Observed freq. | 10 | 27 | 5 | 23 | 21 | 13 | 14 | 19 | 25 | 29 | 24 | 82 | 32 | 13 | 40 | 28 |
| Expected freq. | 11.3 | 22.1 | 9.1 | 30.0 | 42.8 | 22.2 | 24.7 | 30.2 | 24.4 | 25.9 | 21.2 | 50.3 | 35.8 | 9.5 | 25.4 | 20.2 |
| *z* | -0.4 | 1.2 | -1.5 | -1.5 | -3.9^***^ | -2.2^*^ | -2.4^*^ | -2.3^*^ | 0.1 | 0.7 | 0.7 | 5.2^***^ | -0.7 | 1.3 | 3.3^***^ | 1.9 |
| 30-39 |  |  |  |  |  |  |  |  |  |  |  |  |  |  |  |  |
| Observed freq. | 8 | 26 | 5 | 31 | 26 | 14 | 19 | 17 | 21 | 34 | 24 | 74 | 37 | 12 | 26 | 27 |
| Expected freq. | 11.2 | 21.8 | 9.0 | 29.7 | 42.4 | 22.0 | 24.5 | 29.9 | 24.1 | 25.6 | 21.0 | 49.8 | 35.45 | 9.4 | 25.1 | 20.0 |
| *z* | -1.1 | 1.0 | -1.5 | 0.3 | -2.9^**^ | -1.9 | -1.2 | -2.7^**^ | -0.4 | 1.9 | 0.7 | 4.0^***^ | 0.3 | 1.0 | 0.2 | 1.7 |
| 40-49 |  |  |  |  |  |  |  |  |  |  |  |  |  |  |  |  |
| Observed freq. | 18 | 22 | 13 | 30 | 32 | 21 | 16 | 31 | 28 | 25 | 22 | 46 | 35 | 9 | 25 | 17 |
| Expected freq. | 10.9 | 21.2 | 8.8 | 28.9 | 41.2 | 21.4 | 23.8 | 29.1 | 23.5 | 24.9 | 20.4 | 48.4 | 34.5 | 9.1 | 24.4 | 19.5 |
| *z* | 2.4^*^ | 0.2 | 1.6 | 0.2 | -1.7 | -0.1 | -1.8 | 0.4 | 1.1 | 0.0 | 0.4 | -0.4 | 0.1 | 0.0 | 0.1 | -0.6 |
| 50-59 |  |  |  |  |  |  |  |  |  |  |  |  |  |  |  |  |
| Observed freq. | 13 | 19 | 10 | 34 | 50 | 25 | 23 | 24 | 18 | 23 | 16 | 30 | 39 | 4 | 17 | 15 |
| Expected freq. | 10.0 | 19.6 | 8.1 | 26.7 | 38.0 | 19.8 | 22.0 | 26.8 | 21.7 | 23.0 | 18.9 | 44.7 | 31.8 | 8.4 | 22.6 | 18.0 |
| *z* | 1.0 | -0.2 | 0.7 | 1.6 | 2.2^*^ | 1.3 | 0.2 | -0.6 | -0.9 | 0.0 | -0.7 | -2.5^*^ | 1.4 | -1.7 | -1.3 | -0.8 |
| 60-69 |  |  |  |  |  |  |  |  |  |  |  |  |  |  |  |  |
| Observed freq. | 12 | 16 | 16 | 23 | 51 | 19 | 31 | 38 | 21 | 18 | 16 | 27 | 25 | 9 | 12 | 13 |
| Expected freq. | 9.7 | 18.9 | 7.8 | 25.7 | 36.7 | 19.0 | 21.2 | 25.9 | 20.9 | 22.2 | 18.2 | 43.1 | 30.7 | 8.1 | 21.7 | 17.3 |
| *z* | 0.8 | -0.7 | 3.2^**^ | -0.6 | 2.7^**^ | 0.0 | 2.4^*^ | 2.7^**^ | 0.0 | -1.0 | -0.6 | -2.8^**^ | -1.2 | 0.3 | -2.3^*^ | -1.2 |
| 70+ |  |  |  |  |  |  |  |  |  |  |  |  |  |  |  |  |
| Observed freq. | 3 | 20 | 5 | 32 | 59 | 34 | 41 | 44 | 14 | 11 | 8 | 10 | 33 | 5 | 12 | 10 |
| Expected freq. | 9.5 | 18.6 | 7.7 | 25.3 | 36.0 | 18.7 | 20.8 | 25.4 | 20.5 | 21.8 | 17.9 | 42.3 | 30.2 | 8.0 | 21.4 | 17.0 |
| *z* | -2.3^*^ | 0.4 | -1.1 | 1.5 | 4.4^***^ | 3.9^***^ | 4.9^***^ | 4.1^***^ | -1.6 | -2.6^**^ | -2.6^**^ | -5.7^***^ | 0.6 | -1.1 | -2.3^*^ | -1.9 |
| Note. Std. Residuals are z-scores. *z* ± 1.96 are significant at *p* < .05. *z* ± 2.58 are significant at *p* < .01. *z* ± 3.29 are significant at *p* < .001. | | | | | | | | | | | | | | | | |

| ST2. Observed frequencies, expected frequencies, and standardized residuals (*z*) for the relationship between temperament profile and age cohort  Men | | | | | | | | | | | | | | | | |
| --- | --- | --- | --- | --- | --- | --- | --- | --- | --- | --- | --- | --- | --- | --- | --- | --- |
|  | Independent | | Reliable | | Methodical | | Cautious | | Adventurous | | Passionate | | Explosive | | Sensitive | |
|  | nhrp | nhrP | nhRp | nhRP | nHrp | nHrP | nHRp | nHRP | Nhrp | NhrP | NhRp | NhRP | NHrp | NHrP | NHRp | NHRP |
| 17-19 |  |  |  |  |  |  |  |  |  |  |  |  |  |  |  |  |
| Observed freq. | 4 | 0 | 1 | 4 | 7 | 3 | 2 | 4 | 17 | 10 | 6 | 16 | 10 | 1 | 3 | 4 |
| Expected freq. | 3.7 | 7.2 | 2.5 | 8.1 | 9.7 | 4.5 | 3.9 | 5.2 | 7.9 | 8.1 | 4.5 | 10.6 | 7.9 | 1.9 | 3.7 | 2.5 |
| *z* | 0.2 | -2.9 | -1.0 | -1.6 | -1.0 | -0.8 | -1.0 | -0.5 | 3.5 | 0.7 | 0.7 | 1.8 | 0.8 | -0.7 | -0.4 | 1.0 |
| 20-29 |  |  |  |  |  |  |  |  |  |  |  |  |  |  |  |  |
| Observed freq. | 7 | 22 | 1 | 14 | 11 | 6 | 7 | 7 | 16 | 22 | 11 | 36 | 15 | 3 | 13 | 6 |
| Expected freq. | 7.8 | 15.5 | 5.4 | 17.4 | 20.8 | 9.7 | 8.3 | 11.1 | 17.0 | 17.4 | 9.7 | 22.8 | 16.8 | 4.1 | 7.8 | 5.4 |
| *z* | -0.3 | 1.9 | -2.1 | -0.9 | -2.5 | -1.3 | -0.5 | -1.4 | -0.3 | 1.3 | 0.5 | 3.2 | -0.5 | -0.6 | 2.1 | 0.3 |
| 30-39 |  |  |  |  |  |  |  |  |  |  |  |  |  |  |  |  |
| Observed freq. | 4 | 16 | 3 | 14 | 16 | 7 | 4 | 6 | 17 | 21 | 9 | 34 | 17 | 5 | 12 | 9 |
| Expected freq. | 7.7 | 15.2 | 5.4 | 17.1 | 20.4 | 9.5 | 8.2 | 10.9 | 16.8 | 17.1 | 9.5 | 22.4 | 16.6 | 4.0 | 7.7 | 5.4 |
| *z* | -1.5 | 0.2 | -1.1 | -0.9 | -1.1 | -0.9 | -1.6 | -1.7 | 0.1 | 1.1 | -0.2 | 2.8 | 0.1 | 0.5 | 1.7 | 1.7 |
| 40-49 |  |  |  |  |  |  |  |  |  |  |  |  |  |  |  |  |
| Observed freq. | 13 | 13 | 4 | 17 | 15 | 7 | 8 | 12 | 19 | 10 | 11 | 21 | 14 | 7 | 3 | 4 |
| Expected freq. | 7.1 | 14.0 | 4.9 | 15.7 | 18.8 | 8.8 | 7.5 | 10.0 | 15.4 | 15.7 | 8.8 | 20.6 | 15.2 | 3.7 | 7.1 | 4.9 |
| *z* | 2.5 | -0.3 | -0.5 | 0.4 | -1.0 | -0.7 | 0.2 | 0.7 | 1.1 | -1.6 | 0.8 | 0.1 | -0.4 | 1.9 | -1.7 | -0.5 |
| 50-59 |  |  |  |  |  |  |  |  |  |  |  |  |  |  |  |  |
| Observed freq. | 8 | 13 | 7 | 22 | 18 | 8 | 6 | 11 | 9 | 21 | 9 | 10 | 15 | 1 | 7 | 2 |
| Expected freq. | 6.6 | 13.1 | 4.6 | 14.7 | 17.6 | 8.2 | 7.1 | 9.4 | 14.4 | 14.7 | 8.2 | 19.3 | 14.3 | 3.5 | 6.6 | 4.6 |
| *z* | 0.6 | 0.0 | 1.2 | 2.2 | 0.1 | -0.1 | -0.4 | 0.6 | -1.6 | 1.9 | 0.3 | -2.4 | 0.2 | -1.4 | 0.2 | -1.3 |
| 60-69 |  |  |  |  |  |  |  |  |  |  |  |  |  |  |  |  |
| Observed freq. | 8 | 11 | 11 | 9 | 31 | 7 | 11 | 10 | 15 | 12 | 6 | 13 | 11 | 3 | 5 | 4 |
| Expected freq. | 6.6 | 13.1 | 4.6 | 14.7 | 17.6 | 8.2 | 7.1 | 9.4 | 14.4 | 14.7 | 8.2 | 19.3 | 14.3 | 3.5 | 6.6 | 4.6 |
| *z* | 0.6 | -0.7 | 3.3 | -1.7 | 3.7 | -0.5 | 1.6 | 0.2 | 0.2 | -0.8 | -0.9 | -1.7 | -1.0 | -0.3 | -0.7 | -0.3 |
| 70+ |  |  |  |  |  |  |  |  |  |  |  |  |  |  |  |  |
| Observed freq. | 2 | 16 | 5 | 22 | 24 | 19 | 11 | 15 | 7 | 6 | 5 | 4 | 17 | 4 | 3 | 3 |
| Expected freq. | 6.5 | 12.8 | 4.5 | 14.4 | 17.2 | 8.0 | 6.9 | 9.1 | 14.1 | 14.4 | 8.0 | 18.9 | 13.9 | 3.4 | 6.5 | 4.5 |
| *z* | -1.9 | 1.0 | 0.3 | 2.3 | 1.9 | 4.3 | 1.7 | 2.1 | -2.1 | -2.5 | -1.2 | -3.9 | 0.9 | 0.4 | -1.5 | -0.8 |
| Note. Std. Residuals are z-scores. *z* ± 1.96 are significant at *p* < .05. *z* ± 2.58 are significant at *p* < .01. *z* ± 3.29 are significant at *p* < .001. | | | | | | | | | | | | | | | | |

| ST3. Observed frequencies, expected frequencies, and standardized residuals (*z*) for the relationship between temperament profile and age cohort  Women | | | | | | | | | | | | | | | | |
| --- | --- | --- | --- | --- | --- | --- | --- | --- | --- | --- | --- | --- | --- | --- | --- | --- |
|  | Independent | | Reliable | | Methodical | | Cautious | | Adventurous | | Passionate | | Explosive | | Sensitive | |
|  | nhrp | nhrP | nhRp | nhRP | nHrp | nHrP | nHRp | nHRP | Nhrp | NhrP | NhRp | NhRP | NHrp | NHrP | NHRp | NHRP |
| 17-19 |  |  |  |  |  |  |  |  |  |  |  |  |  |  |  |  |
| Observed freq. | 0 | 3 | 0 | 4 | 12 | 5 | 3 | 5 | 3 | 6 | 12 | 18 | 5 | 4 | 18 | 7 |
| Expected freq. | 1.8 | 3.4 | 1.9 | 6.5 | 11.1 | 6.3 | 8.2 | 9.6 | 3.8 | 4.4 | 5.8 | 13.8 | 9.6 | 2.7 | 8.8 | 7.3 |
| *z* | -1.4 | -0.3 | -1.4 | -1.0 | 0.3 | -0.6 | -2.0 | -1.6 | -0.5 | 0.8 | 2.8 | 1.3 | -1.6 | 0.8 | 3.4 | -0.1 |
| 20-29 |  |  |  |  |  |  |  |  |  |  |  |  |  |  |  |  |
| Observed freq. | 3 | 5 | 4 | 9 | 10 | 7 | 7 | 12 | 9 | 7 | 13 | 46 | 17 | 10 | 27 | 22 |
| Expected freq. | 3.6 | 6.8 | 3.7 | 12.8 | 22.0 | 12.5 | 16.2 | 19.0 | 7.6 | 8.8 | 11.5 | 27.4 | 19.0 | 5.3 | 17.3 | 14.4 |
| *z* | -0.3 | -0.8 | 0.2 | -1.2 | -3.0 | -1.7 | -2.6 | -1.8 | 0.6 | -0.7 | 0.5 | 4.2 | -0.5 | 2.2 | 2.6 | 2.3 |
| 30-39 |  |  |  |  |  |  |  |  |  |  |  |  |  |  |  |  |
| Observed freq. | 4 | 10 | 2 | 17 | 10 | 7 | 15 | 11 | 4 | 13 | 15 | 40 | 20 | 7 | 14 | 18 |
| Expected freq. | 3.5 | 6.8 | 3.7 | 12.7 | 21.9 | 12.4 | 16.1 | 18.9 | 7.6 | 8.7 | 11.5 | 27.3 | 18.9 | 5.3 | 17.3 | 14.4 |
| *z* | 0.3 | 1.4 | -1.0 | 1.3 | -2.9 | -1.7 | -0.3 | -2.1 | -1.4 | 1.6 | 1.2 | 2.9 | 0.3 | 0.8 | -0.9 | 1.1 |
| 40-49 |  |  |  |  |  |  |  |  |  |  |  |  |  |  |  |  |
| Observed freq. | 5 | 9 | 9 | 13 | 17 | 14 | 8 | 19 | 9 | 15 | 11 | 25 | 21 | 2 | 22 | 13 |
| Expected freq. | 3.6 | 6.9 | 3.8 | 13.1 | 22.5 | 12.7 | 16.5 | 19.3 | 7.8 | 8.9 | 11.7 | 27.9 | 19.3 | 5.5 | 17.7 | 14.7 |
| *z* | 0.8 | 0.9 | 2.9 | 0.0 | -1.3 | 0.4 | -2.4 | -0.1 | 0.5 | 2.3 | -0.2 | -0.7 | 0.4 | -1.6 | 1.2 | -0.5 |
| 50-59 |  |  |  |  |  |  |  |  |  |  |  |  |  |  |  |  |
| Observed freq. | 5 | 6 | 3 | 12 | 32 | 17 | 17 | 13 | 9 | 2 | 7 | 20 | 24 | 3 | 10 | 13 |
| Expected freq. | 3.3 | 6.3 | 3.5 | 11.9 | 20.5 | 11.6 | 15.0 | 17.6 | 7.1 | 8.1 | 10.7 | 25.4 | 17.6 | 5.0 | 16.1 | 13.4 |
| *z* | 1.0 | -0.1 | -0.3 | 0.0 | 2.9 | 1.8 | 0.6 | -1.2 | 0.8 | -2.4 | -1.3 | -1.3 | 1.7 | -1.0 | -1.7 | -0.1 |
| 60-69 |  |  |  |  |  |  |  |  |  |  |  |  |  |  |  |  |
| Observed freq. | 4 | 5 | 5 | 14 | 20 | 12 | 20 | 28 | 6 | 6 | 10 | 14 | 14 | 6 | 7 | 9 |
| Expected freq. | 3.1 | 5.9 | 3.2 | 11.1 | 19.1 | 10.8 | 14.0 | 16.4 | 6.6 | 7.6 | 10.0 | 23.7 | 16.4 | 4.6 | 15.0 | 12.5 |
| *z* | 0.6 | -0.4 | 1.1 | 1.0 | 0.2 | 0.4 | 1.8 | 3.2 | -0.3 | -0.6 | 0.0 | -2.3 | -0.7 | 0.7 | -2.3 | -1.1 |
| 70+ |  |  |  |  |  |  |  |  |  |  |  |  |  |  |  |  |
| Observed freq. | 1 | 4 | 0 | 10 | 35 | 15 | 30 | 29 | 7 | 5 | 3 | 6 | 16 | 1 | 9 | 7 |
| Expected freq. | 3.1 | 5.8 | 3.2 | 11.0 | 18.9 | 10.7 | 13.9 | 16.2 | 6.5 | 7.5 | 9.9 | 23.4 | 16.2 | 4.6 | 14.8 | 12.3 |
| *z* | -1.3 | -0.8 | -1.9 | -0.3 | 4.2 | 1.5 | 4.9 | 3.6 | 0.2 | -1.0 | -2.4 | -4.2 | -0.1 | -1.8 | -1.7 | -1.7 |
| Note. Std. Residuals are z-scores. *z* ± 1.96 are significant at *p* < .05. *z* ± 2.58 are significant at *p* < .01. *z* ± 3.29 are significant at *p* < .001. | | | | | | | | | | | | | | | | |

| ST4. Observed frequencies, expected frequencies, and standardized residuals (*z*) for the relationship between character profile and age cohort  Full sample | | | | | | | | |
| --- | --- | --- | --- | --- | --- | --- | --- | --- |
|  | Apathetic | Disorganized | Dependent | Moody | Bossy | Absolutist | Organized | Creative |
|  | sct | scT | sCt | sCT | Sct | ScT | SCt | SCT |
| 17-19 |  |  |  |  |  |  |  |  |
| Observed freq. | 49 | 43 | 14 | 25 | 15 | 8 | 21 | 23 |
| Expected freq. | 31.1 | 35.8 | 10.9 | 20.0 | 17.1 | 9.6 | 33.1 | 40.4 |
| *z* | 3.6*** | 1.4 | 1.0 | 1.2 | -0.6 | -0.6 | -2.4** | -3.2** |
| 20-29 |  |  |  |  |  |  |  |  |
| Observed freq. | 63 | 70 | 32 | 42 | 39 | 18 | 79 | 62 |
| Expected freq. | 63.7 | 73.1 | 22.2 | 41.0 | 35.0 | 19.7 | 67.7 | 82.6 |
| *z* | -0.1 | -0.4 | 2.3** | 0.2 | 0.8 | -0.4 | 1.7 | -2.8** |
| 30-39 |  |  |  |  |  |  |  |  |
| Observed freq. | 57 | 66 | 23 | 31 | 36 | 13 | 83 | 92 |
| Expected freq. | 63.1 | 72.4 | 22.0 | 40.6 | 34.6 | 19.5 | 67.0 | 81.8 |
| *z* | -0.9 | -0.9 | 0.2 | -1.7 | 0.3 | -1.7 | 2.3* | 1.4 |
| 40-49 |  |  |  |  |  |  |  |  |
| Observed freq. | 55 | 48 | 24 | 30 | 44 | 18 | 85 | 86 |
| Expected freq. | 61.3 | 70.4 | 21.4 | 39.4 | 33.7 | 19.0 | 65.2 | 79.5 |
| *z* | -1.0 | -3.2** | 0.6 | -1.7 | 2.0* | -0.3 | 2.9** | 0.9 |
| 50-59 |  |  |  |  |  |  |  |  |
| Observed freq. | 65 | 71 | 13 | 32 | 34 | 19 | 49 | 77 |
| Expected freq. | 56.6 | 65.0 | 19.8 | 36.4 | 31.1 | 17.5 | 60.1 | 73.4 |
| *z* | 1.3 | 0.9 | -1.7 | -0.8 | 0.6 | 0.4 | -1.7 | 0.5 |
| 60-69 |  |  |  |  |  |  |  |  |
| Observed freq. | 49 | 72 | 15 | 35 | 27 | 15 | 56 | 78 |
| Expected freq. | 54.6 | 62.8 | 19.0 | 35.1 | 30.0 | 16.9 | 58.0 | 70.8 |
| *z* | -0.9 | 1.4 | -1.0 | 0.0 | -0.6 | -0.5 | -0.3 | 1.0 |
| 70+ |  |  |  |  |  |  |  |  |
| Observed freq. | 46 | 71 | 13 | 52 | 16 | 28 | 35 | 80 |
| Expected freq. | 53.6 | 61.6 | 18.7 | 34.5 | 29.5 | 16.6 | 57.0 | 69.5 |
| *z* | -1.2 | 1.4 | -1.5 | 3.4*** | -2.8** | 3.1** | -3.4*** | 1.5 |

| ST5. Observed frequencies, expected frequencies, and standardized residuals (*z*) for the relationship between character profile and age cohort  Men | | | | | | | | |
| --- | --- | --- | --- | --- | --- | --- | --- | --- |
|  | Apathetic | Disorganized | Dependent | Moody | Bossy | Absolutist | Organized | Creative |
|  | sct | scT | sCt | sCT | Sct | ScT | SCt | SCT |
| 17-19 |  |  |  |  |  |  |  |  |
| Observed freq. | 21 | 25 | 5 | 6 | 8 | 5 | 15 | 7 |
| Expected freq. | 17.5 | 17.2 | 4.4 | 5.8 | 10.0 | 4.9 | 16.1 | 16.0 |
| *z* | 1.0 | 2.2 | 0.3 | 0.1 | -0.7 | 0.0 | -0.3 | -2.6 |
| 20-29 |  |  |  |  |  |  |  |  |
| Observed freq. | 45 | 32 | 16 | 12 | 23 | 6 | 33 | 30 |
| Expected freq. | 37.4 | 36.9 | 9.5 | 12.4 | 21.4 | 10.5 | 34.5 | 34.2 |
| *z* | 1.5 | -1.0 | 2.4 | -0.1 | 0.4 | -1.6 | -0.3 | -0.9 |
| 30-39 |  |  |  |  |  |  |  |  |
| Observed freq. | 30 | 37 | 12 | 11 | 25 | 7 | 42 | 30 |
| Expected freq. | 36.9 | 36.4 | 9.4 | 12.2 | 21.1 | 10.4 | 34.0 | 33.7 |
| *z* | -1.4 | 0.1 | 1.0 | -0.4 | 1.0 | -1.2 | 1.7 | -0.8 |
| 40-49 |  |  |  |  |  |  |  |  |
| Observed freq. | 36 | 22 | 11 | 10 | 22 | 11 | 34 | 32 |
| Expected freq. | 33.8 | 33.4 | 8.6 | 11.2 | 19.4 | 9.5 | 31.2 | 30.9 |
| *z* | 0.5 | -2.4 | 0.9 | -0.4 | 0.7 | 0.5 | 0.6 | 0.2 |
| 50-59 |  |  |  |  |  |  |  |  |
| Observed freq. | 33 | 35 | 3 | 9 | 20 | 9 | 29 | 29 |
| Expected freq. | 31.7 | 31.3 | 8.1 | 10.5 | 18.2 | 8.9 | 29.3 | 29.0 |
| *z* | 0.3 | 0.8 | -2.0 | -0.5 | 0.5 | 0.0 | -0.1 | 0.0 |
| 60-69 |  |  |  |  |  |  |  |  |
| Observed freq. | 32 | 32 | 6 | 6 | 19 | 9 | 28 | 35 |
| Expected freq. | 31.7 | 31.3 | 8.1 | 10.5 | 18.2 | 8.9 | 29.3 | 29.0 |
| *z* | 0.1 | 0.2 | -0.8 | -1.6 | 0.2 | 0.0 | -0.3 | 1.3 |
| 70+ |  |  |  |  |  |  |  |  |
| Observed freq. | 23 | 34 | 3 | 19 | 9 | 15 | 22 | 38 |
| Expected freq. | 31.0 | 30.5 | 7.9 | 10.3 | 17.7 | 8.7 | 28.6 | 28.3 |
| *z* | -1.7 | 0.7 | -1.9 | 3.0 | -2.4 | 2.4 | -1.5 | 2.2 |

| ST6. Observed frequencies, expected frequencies, and standardized residuals (*z*) for the relationship between character profile and age cohort  Women | | | | | | | | |
| --- | --- | --- | --- | --- | --- | --- | --- | --- |
|  | Apathetic | Disorganized | Dependent | Moody | Bossy | Absolutist | Organized | Creative |
|  | sct | scT | sCt | sCT | Sct | ScT | SCt | SCT |
| 17-19 |  |  |  |  |  |  |  |  |
| Observed freq. | 28 | 17 | 9 | 19 | 7 | 3 | 6 | 16 |
| Expected freq. | 13.4 | 18.3 | 6.4 | 14.2 | 7.0 | 4.7 | 16.8 | 24.3 |
| *z* | 4.4 | -0.3 | 1.1 | 1.4 | 0.0 | -0.8 | -3.0 | -2.0 |
| 20-29 |  |  |  |  |  |  |  |  |
| Observed freq. | 18 | 38 | 16 | 30 | 16 | 12 | 46 | 32 |
| Expected freq. | 26.6 | 36.2 | 12.6 | 28.2 | 13.8 | 9.2 | 33.2 | 48.1 |
| *z* | -1.9 | 0.4 | 1.1 | 0.4 | 0.7 | 1.0 | 2.6 | -2.9 |
| 30-39 |  |  |  |  |  |  |  |  |
| Observed freq. | 27 | 29 | 11 | 20 | 11 | 6 | 41 | 62 |
| Expected freq. | 26.5 | 36.0 | 12.6 | 28.1 | 13.7 | 9.2 | 33.1 | 47.9 |
| *z* | 0.1 | -1.4 | -0.5 | -1.8 | -0.8 | -1.2 | 1.6 | 2.5 |
| 40-49 |  |  |  |  |  |  |  |  |
| Observed freq. | 19 | 26 | 13 | 20 | 22 | 7 | 51 | 54 |
| Expected freq. | 27.1 | 36.8 | 12.9 | 28.8 | 14.0 | 9.4 | 33.9 | 49.1 |
| *z* | -1.8 | -2.2 | 0.0 | -1.9 | 2.4 | -0.9 | 3.5 | 0.9 |
| 50-59 |  |  |  |  |  |  |  |  |
| Observed freq. | 32 | 36 | 10 | 23 | 14 | 10 | 20 | 48 |
| Expected freq. | 24.7 | 33.5 | 11.7 | 26.2 | 12.8 | 8.6 | 30.8 | 44.7 |
| *z* | 1.7 | 0.5 | -0.6 | -0.7 | 0.4 | 0.5 | -2.3 | 0.6 |
| 60-69 |  |  |  |  |  |  |  |  |
| Observed freq. | 17 | 40 | 9 | 29 | 8 | 6 | 28 | 43 |
| Expected freq. | 23.0 | 31.3 | 10.9 | 24.4 | 11.9 | 8.0 | 28.8 | 41.7 |
| *z* | -1.4 | 1.8 | -0.7 | 1.1 | -1.3 | -0.8 | -0.2 | 0.3 |
| 70+ |  |  |  |  |  |  |  |  |
| Observed freq. | 23 | 37 | 10 | 33 | 7 | 13 | 13 | 42 |
| Expected freq. | 22.8 | 30.9 | 10.8 | 24.1 | 11.8 | 7.9 | 28.4 | 41.2 |
| *z* | 0.1 | 1.3 | -0.3 | 2.1 | -1.6 | 2.0 | -3.4 | 0.2 |

| ST7. Observed frequencies, expected frequencies, and standardized residuals (*z*) for the relationship between phenotypic network and age cohort  Full sample | | | |
| --- | --- | --- | --- |
|  | Emotional-Unreliable | Organized-Reliable | Creative-Reliable |
| 17-19 |  |  |  |
| Observed freq. | 131 | 44 | 23 |
| Expected freq. | 97.8 | 59.8 | 40.4 |
| *z* | 4.9^***^ | -2.6^**^ | -3.2^**^ |
| 20-29 |  |  |  |
| Observed freq. | 207 | 136 | 62 |
| Expected freq. | 200.0 | 122.4 | 82.6 |
| *z* | 0.8 | 1.6 | -2.8^**^ |
| 30-39 |  |  |  |
| Observed freq. | 177 | 132 | 92 |
| Expected freq. | 198.0 | 121.2 | 81.8 |
| *z* | -2.3^*^ | 1.3 | 1.4 |
| 40-49 |  |  |  |
| Observed freq. | 157 | 147 | 86 |
| Expected freq. | 192.6 | 117.9 | 79.5 |
| *z* | -3.9^***^ | 3.5^***^ | 0.9 |
| 50-59 |  |  |  |
| Observed freq. | 181 | 102 | 77 |
| Expected freq. | 177.8 | 108.8 | 73.4 |
| *z* | 0.4 | -0.8 | 0.5 |
| 60-69 |  |  |  |
| Observed freq. | 171 | 98 | 78 |
| Expected freq. | 171.4 | 104.9 | 70.8 |
| *z* | 0.0 | -0.9 | 1.0 |
| 70+ |  |  |  |
| Observed freq. | 182 | 79 | 80 |
| Expected freq. | 168.4 | 103.1 | 69.4 |
| *z* | 1.6 | -3.1^**^ | 1.5 |

| ST8. Observed frequencies, expected frequencies, and standardized residuals (*z*) for the relationship between phenotypic network and age cohort  Men | | | |
| --- | --- | --- | --- |
|  | Emotional-Unreliable | Organized-Reliable | Creative-Reliable |
| 17-19 |  |  |  |
| Observed freq. | 57 | 28 | 7 |
| Expected freq. | 45.0 | 31.1 | 16.0 |
| *z* | 2.6 | -0.7 | -2.6 |
| 20-29 |  |  |  |
| Observed freq. | 105 | 62 | 30 |
| Expected freq. | 96.3 | 66.5 | 34.2 |
| *z* | 1.4 | -0.7 | -0.9 |
| 30-39 |  |  |  |
| Observed freq. | 90 | 74 | 30 |
| Expected freq. | 94.8 | 65.5 | 33.7 |
| *z* | -0.8 | 1.4 | -0.8 |
| 40-49 |  |  |  |
| Observed freq. | 79 | 67 | 32 |
| Expected freq. | 87.0 | 60.1 | 30.9 |
| *z* | -1.3 | 1.2 | 0.2 |
| 50-59 |  |  |  |
| Observed freq. | 80 | 58 | 29 |
| Expected freq. | 81.6 | 56.4 | 29.0 |
| *z* | -0.3 | 0.3 | 0.0 |
| 60-69 |  |  |  |
| Observed freq. | 76 | 56 | 35 |
| Expected freq. | 81.6 | 56.4 | 29.0 |
| *z* | -0.9 | -0.1 | 1.3 |
| 70+ |  |  |  |
| Observed freq. | 79 | 46 | 38 |
| Expected freq. | 79.7 | 55.0 | 28.3 |
| *z* | -0.1 | -1.6 | 2.2 |

| ST9. Observed frequencies, expected frequencies, and standardized residuals (*z*) for the relationship between phenotypic network and age cohort  Women | | | |
| --- | --- | --- | --- |
|  | Emotional-Unreliable | Organized-Reliable | Creative-Reliable |
| 17-19 |  |  |  |
| Observed freq. | 73 | 16 | 16 |
| Expected freq. | 52.3 | 28.4 | 24.3 |
| *z* | 4.2 | -2.8 | -2.0 |
| 20-29 |  |  |  |
| Observed freq. | 102 | 74 | 32 |
| Expected freq. | 103.6 | 56.3 | 48.1 |
| *z* | -0.2 | 3.0 | -2.9 |
| 30-39 |  |  |  |
| Observed freq. | 87 | 58 | 62 |
| Expected freq. | 103.1 | 56.0 | 47.9 |
| *z* | -2.4 | 0.3 | 2.5 |
| 40-49 |  |  |  |
| Observed freq. | 78 | 80 | 54 |
| Expected freq. | 105.6 | 57.3 | 49.1 |
| *z* | -4.1 | 3.8 | 0.9 |
| 50-59 |  |  |  |
| Observed freq. | 101 | 44 | 48 |
| Expected freq. | 96.1 | 52.2 | 44.7 |
| *z* | 0.8 | -1.4 | 0.6 |
| 60-69 |  |  |  |
| Observed freq. | 95 | 42 | 43 |
| Expected freq. | 89.6 | 48.7 | 41.7 |
| *z* | 0.9 | -1.2 | 0.3 |
| 70+ |  |  |  |
| Observed freq. | 103 | 33 | 42 |
| Expected freq. | 88.7 | 48.1 | 41.2 |
| *z* | 2.3 | -2.8 | 0.2 |

| ST10. Observed frequencies, expected frequencies, and standardized residuals (*z*) for the relationship between temperament profile and gender | | | | | | | | | | | | | | | | |
| --- | --- | --- | --- | --- | --- | --- | --- | --- | --- | --- | --- | --- | --- | --- | --- | --- |
|  | Independent | | Reliable | | Methodical | | Cautious | | Adventurous | | Passionate | | Explosive | | Sensitive | |
|  | nhrp | nhrP | nhRp | nhRP | nHrp | nHrP | nHRp | nHRP | Nhrp | NhrP | NhRp | NhRP | NHrp | NHrP | NHRp | NHRP |
| Men |  |  |  |  |  |  |  |  |  |  |  |  |  |  |  |  |
| Observed freq. | 46 | 91 | 32 | 102 | 122 | 57 | 49 | 65 | 100 | 102 | 57 | 134 | 99 | 24 | 46 | 32 |
| Expected freq. | 32.3 | 63.1 | 26.1 | 85.9 | 122.4 | 63.6 | 70.7 | 86.3 | 69.7 | 74.0 | 60.7 | 143.7 | 102.5 | 27.0 | 72.6 | 57.4 |
| *z* | 3.4 | 5.0 | 1.6 | 2.5 | -0.1 | -1.2 | -3.7 | -3.3 | 5.2 | 4.6 | -0.7 | -1.2 | -0.5 | -0.8 | -4.4 | -4.7 |
| Women |  |  |  |  |  |  |  |  |  |  |  |  |  |  |  |  |
| Observed freq. | 22 | 42 | 23 | 79 | 136 | 77 | 100 | 117 | 47 | 54 | 71 | 169 | 117 | 33 | 107 | 89 |
| Expected freq. | 35.7 | 69.9 | 28.9 | 95.1 | 135.6 | 70.4 | 78.3 | 95.7 | 77.3 | 82.0 | 67.3 | 159.3 | 113.5 | 30.0 | 80.4 | 63.6 |
| *z* | -3.4 | -5.0 | -1.6 | -2.5 | 0.1 | 1.2 | 3.7 | 3.3 | -5.2 | -4.6 | 0.7 | 1.2 | 0.5 | 0.8 | 4.4 | 4.7 |

| ST11. Observed frequencies, expected frequencies, and standardized residuals (*z*) for the relationship between character profile and gender | | | | | | | | |
| --- | --- | --- | --- | --- | --- | --- | --- | --- |
|  | Apathetic | Disorganized | Dependent | Moody | Bossy | Absolutist | Organized | Creative |
|  | sct | scT | sCt | sCT | Sct | ScT | SCt | SCT |
| Men |  |  |  |  |  |  |  |  |
| Observed freq. | 220 | 217 | 56 | 73 | 126 | 62 | 203 | 201 |
| Expected freq. | 182.2 | 208.7 | 63.6 | 117.2 | 100.1 | 56.5 | 193.6 | 236.2 |
| *z* | 4.2 | 0.9 | -1.3 | -5.9 | 3.7 | 1.0 | 1.0 | -3.5 |
| Women |  |  |  |  |  |  |  |  |
| Observed freq. | 164 | 223 | 78 | 174 | 85 | 57 | 205 | 297 |
| Expected freq. | 201.8 | 231.3 | 70.4 | 129.8 | 110.9 | 625 | 214.4 | 261.8 |
| *z* | -4.2 | -0.9 | 1.3 | 5.9 | -3.7 | -1.0 | -1.0 | 3.5 |

| ST12. Observed frequencies, expected frequencies, and standardized residuals (*z*) for the relationship between phenotypic network and gender | | | |
| --- | --- | --- | --- |
|  | Emotional-Unreliable | Organized-Reliable | Creative-Reliable |
| Men |  |  |  |
| Observed freq. | 566 | 391 | 201 |
| Expected freq. | 571.6 | 350.1 | 236.2 |
| *z* | -0.5 | 3.6 | -3.5 |
| Women |  |  |  |
| Observed freq. | 639 | 347 | 297 |
| Expected freq. | 633.4 | 387.9 | 261.8 |
| *z* | 0.5 | -3.6 | 3.5 |

| ST13. Distribution of achieved education level for each age cohort | | | | | | | |
| --- | --- | --- | --- | --- | --- | --- | --- |
|  | Age Cohort | | | | | | |
| Level of education | 17-19 | 20-29 | 30-39 | 40-49 | 50-59 | 60-69 | 70+ |
| < 4^th^ grade | 0 | 0 | 0 | 0 | 0 | 6 | 22 |
| 4^th^ grade | 0 | 0 | 3 | 29 | 94 | 153 | 221 |
| 6^th^ grade | 3 | 10 | 15 | 37 | 39 | 36 | 20 |
| 9^th^ grade | 61 | 71 | 54 | 86 | 68 | 46 | 31 |
| 12^th^ grade | 133 | 201 | 130 | 110 | 79 | 33 | 7 |
| Degree | 1 | 109 | 173 | 115 | 67 | 60 | 28 |
| Vocational degree | 0 | 3 | 1 | 1 | 7 | 5 | 2 |
| Master’s degree | 0 | 10 | 22 | 7 | 1 | 3 | 1 |
| PhD | 0 | 0 | 1 | 1 | 2 | 1 | 0 |

| ST14. Full frequency table of temperament profiles, character profiles, and joint temperament-character networks. | | | | | | | | | | | | | | | | | | | | |
| --- | --- | --- | --- | --- | --- | --- | --- | --- | --- | --- | --- | --- | --- | --- | --- | --- | --- | --- | --- | --- |
|  |  | Temperament Profile | | | | | | | | | | | | | | | | |  |  |
|  |  | Independent | | Reliable | | Methodical | | Cautious | | Adventurous | | Passionate | | | Explosive | | Sensitive | |  |  |
| Network | Character  Profile | nhrp | nhrP | nhRp | nhRP | nHrp | nHrP | nHRp | nHRP | Nhrp | NhrP | | NhRp | NhRP | NHrp | NHrP | NHRp | NHRP | C Total | N Total |
| Emo-Un. | sct | 12 | 14 | 2 | 6 | 88 | 20 | 16 | 5 | 40 | 23 | | 13 | 10 | 92 | 9 | 29 | 5 | 384 | 1206 |
|  | scT | 7 | 16 | 2 | 9 | 54 | 38 | 25 | 35 | 36 | 37 | | 13 | 37 | 64 | 19 | 28 | 21 | 441 |  |
|  | sCt | 1 | 3 | 3 | 6 | 26 | 5 | 13 | 13 | 5 | 6 | | 11 | 5 | 11 | 2 | 17 | 7 | 134 |  |
|  | sCT | 3 | 7 | 0 | 9 | 20 | 15 | 22 | 44 | 2 | 2 | | 11 | 22 | 13 | 10 | 29 | 38 | 247 |  |
| Org-Rel. | Sct | 10 | 24 | 1 | 6 | 21 | 5 | 6 | 2 | 31 | 27 | | 15 | 27 | 19 | 6 | 7 | 4 | 211 | 738 |
|  | ScT | 1 | 11 | 2 | 11 | 11 | 12 | 9 | 5 | 4 | 16 | | 6 | 15 | 2 | 3 | 3 | 8 | 119 |  |
|  | SCt | 22 | 31 | 31 | 46 | 20 | 15 | 24 | 33 | 17 | 23 | | 29 | 61 | 13 | 5 | 24 | 14 | 408 |  |
| Cre-Rel. | SCT | 12 | 27 | 14 | 88 | 18 | 24 | 34 | 45 | 12 | 22 | | 30 | 126 | 2 | 3 | 16 | 25 | 498 | 498 |
|  | T Total | 68 | 133 | 55 | 181 | 258 | 134 | 149 | 182 | 147 | 156 | | 128 | 303 | 216 | 57 | 153 | 122 |  |  |
| *Note.* Emo-Un = Emotional-Unreliable Network. Org-Rel = Organized-Reliable Network. Cre-Rel = Creative-Reliable Network. | | | | | | | | | | | | | | | | | | | | |
